# Urolithin A exerts antiobesity effects through enhancing adipose tissue thermogenesis in mice

**DOI:** 10.1371/journal.pbio.3000688

**Published:** 2020-03-27

**Authors:** Bo Xia, Xiao Chen Shi, Bao Cai Xie, Meng Qing Zhu, Yan Chen, Xin Yi Chu, Guo He Cai, Min Liu, Shi Zhen Yang, Grant A. Mitchell, Wei Jun Pang, Jiang Wei Wu

**Affiliations:** 1 Key Laboratory of Animal Genetics, Breeding and Reproduction of Shaanxi Province, College of Animal Science and Technology, Northwest A&F University, Yangling, Shaanxi, China; 2 Division of Medical Genetics, Department of Paediatrics, Université de Montréal and Centre Hospitalier Universitaire Sainte-Justine, Montréal, Québec, Canada; University of California Los Angeles, UNITED STATES

## Abstract

Obesity leads to multiple health problems, including diabetes, fatty liver, and even cancer. Here, we report that urolithin A (UA), a gut-microflora–derived metabolite of pomegranate ellagitannins (ETs), prevents diet-induced obesity and metabolic dysfunctions in mice without causing adverse effects. UA treatment increases energy expenditure (EE) by enhancing thermogenesis in brown adipose tissue (BAT) and inducing browning of white adipose tissue (WAT). Mechanistically, UA-mediated increased thermogenesis is caused by an elevation of triiodothyronine (T3) levels in BAT and inguinal fat depots. This is also confirmed in UA-treated white and brown adipocytes. Consistent with this mechanism, UA loses its beneficial effects on activation of BAT, browning of white fat, body weight control, and glucose homeostasis when thyroid hormone (TH) production is blocked by its inhibitor, propylthiouracil (PTU). Conversely, administration of exogenous tetraiodothyronine (T4) to PTU-treated mice restores UA-induced activation of BAT and browning of white fat and its preventive role on high-fat diet (HFD)-induced weight gain. Together, these results suggest that UA is a potent antiobesity agent with potential for human clinical applications.

## Abstract 2

This study shows that urolithin A, a gut-microflora–derived metabolite of pomegranate ellagitannins, prevents diet-induced obesity and metabolic dysfunction in mice via the thyroid hormone pathway without causing adverse effects.

## Introduction

Natural products are a rich resource for the identification of bioactive compounds with beneficial pharmacological activities [1,2]. Pomegranate fruit, red berries, and walnuts are highly valued as “healthy” food and have been consumed by humans from hunter–gatherer days until the present [3,4]. Among other bioactive compounds, ellagitannins (ETs) have been shown to facilitate many of the beneficial effects of pomegranate. ETs exhibit antioxidant and anti-inflammatory functions [5–10]. Once ingested, ETs are quickly metabolized in the intestinal tract to ellagic acid (EA) [8,11,12]. Successive lactone hydrolysis and decarboxylation, as well as hydroxyl group reduction of ETs by the gut microflora, eventually result in the formation of 5 urolithins [13,14], named urolithin A (UA), urolithin B (UB), urolithin C (UC), urolithin D (UD), and iso-urolithin A (iso-UA).

The species composition of urolithins differs among individuals in response to ET ingestion and seems to affect their bioactivity [15,16]. For example, obese individuals who converted pomegranate or walnut ETs into UA, iso-UA, and/or UB were at higher cardiovascular risk than obese individuals who generated only UA [15]. In addition, aging is a key factor affecting the species-specific formation of urolithins from ETs. With aging, UA formation gradually decreases while UB formation increases, leading to a lower UA/UB ratio [17]. Because the ET conversion to various urolithins is predominantly executed by the gut microflora, the interindividual and age-related differences are thought to depend on the highly variable composition of the microbiome. To avoid imponderables, it is preferable to use distinct urolithins rather than their precursor ETs in bioactivity studies.

Among urolithins, UA shows anticancer, anti-inflammation, and antiaging effects and has demonstrated compelling biological activities [14,18–20]. The direct effects of UA have been studied in different types of cells in vitro, including human prostate and colorectal cancer cells [21,22] and B16 melanoma cells [23]. In these experiments, UA consistently prevented cancer cell proliferation and metastasis, arguing for a potential anticancer effect of UA. In addition, UA inhibits lipopolysaccharide (LPS)-induced inflammation in murine macrophages [24,25]. The anti-inflammatory function of UA was confirmed in the hearts of diabetic rats [26]. In addition, UA efficiently induces mitophagy and prolongs life span in *Caenorhabditis elegans* and increases skeletal muscle function in rodents [27].

Recently, the effect of UA on skeletal muscle function in humans and its safety have been investigated in a clinical trial (NCT04160312). In the study, consumption of UA by a group of 36 people with the mean age of 66 years, in which UA was given by a single dose (a maximum of 1,000 mg per day) for 28 days followed by multiple dosing for another 28 days, was tested along with plasma acylcarnitine and skeletal muscle mitochondrial gene expression. Within the tested dose and period, improved mitochondrial function of skeletal muscle was shown in these people without observing any side effects [28]. Subsequently, FDA had completed its safety evaluation (GRN000791), and the substance received the GRAS (generally recognized as safe) designation from the Food and Drug Administration (FDA).

Recent evidence suggests that urolithins not only affect inflammation, cancer, and longevity but also act on cellular lipid metabolism and adipogenesis. UA, UC, and UD significantly reduced triglyceride (TG) accumulation and increased fatty acid (FA) oxidation in cultured human adipocytes and hepatocytes [29]. Furthermore, UA inhibited TG accumulation and the expression of adipogenesis-related genes in murine 3T3-L1 adipocytes [30]. These findings suggest a potential antiobesity effect of UA. Therefore, we investigated the effects of UA on lipid, glucose, and energy homeostasis. The results were striking: UA improved the metabolic health in high-fat diet (HFD)-treated mice and reduced obesity and hyperglycemia. The underlying mechanism involves a thyroid hormone (TH)-dependent increase in energy expenditure (EE) by enhancing thermogenesis in brown adipose tissue (BAT) and inducing browning of white adipose tissue (WAT).

## Results

### UA prevents both HFD-induced and genetic obesity

**Fig 1:**
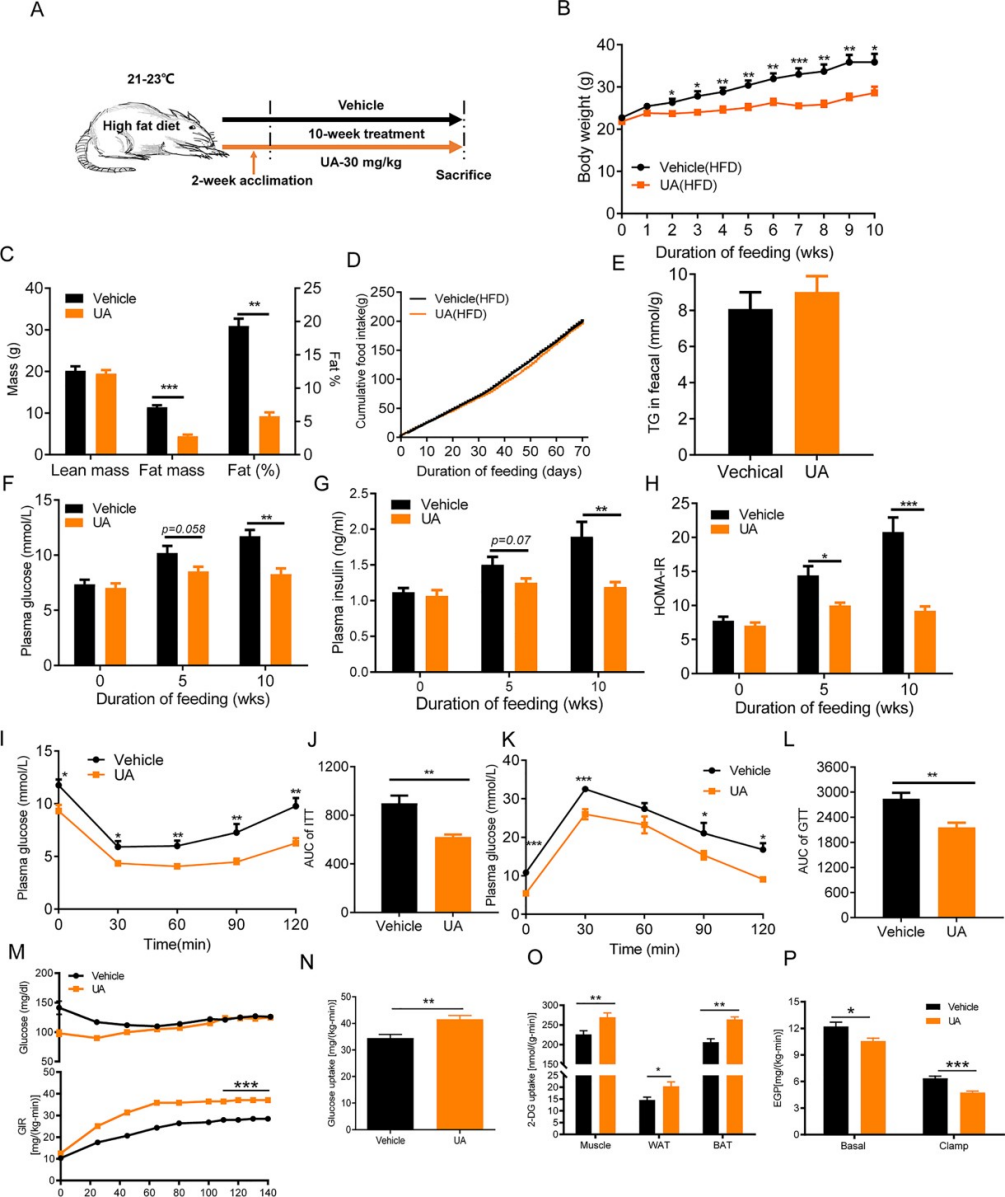
UA prevents HFD-induced obesity and hyperglycemia in mice. (A) Schematic diagram of mice treatment. Six pairs of C57BL/6 male mice fed an HFD were treated with 30 mg/kg/day of UA or vehicle by gavage starting from 8 weeks of age for a period of 10 weeks. (B) Body weight time course. (C) Body composition measured by DEXA scan after 7 weeks of treatment. (D) Cumulative food intake. (E) Levels of fecal TG after 10 weeks of treatment. levels of (F) plasma glucose and (G) insulin after 5 h fasting (*n* = 6). (H) HOMA-IR index. (I) ITT performed after 6 weeks of treatment and (J) the AUC. (K) GTT performed after 8 weeks of treatment and (L) the AUC. (M) Time course of plasma glucose and GIR during the hyperinsulinemic–euglycemic clamp. (N) Whole-body insulin-stimulated glucose uptake. (O) Insulin-stimulated 2-DG uptake in skeletal muscle, BAT, and WAT. (P) Basal and clamp EGP. The underlying data for this figure can be found in S1 Data. AUC, area under the curve; BAT, brown adipose tissue; DEXA, dual-energy X-ray absorptiometry; EGP, endogenous glucose production; GIR, glucose infusion rate; GTT, glucose tolerance test; HFD, high-fat diet; HOMA-IR, homeostasis model assessment-estimated insulin resistance; ITT, insulin tolerance test; TG, triglyceride; UA, urolithin A; WAT, white adipose tissue; 2-DG, 2-deoxyglucose.

To investigate the effect of UA on body weight, male C57BL/6 mice fed an HFD were treated with 30 mg/kg/day of UA or vehicle by gavage starting from 8 weeks of age (Fig 1A). Compared with vehicle-treated controls, UA-treated mice showed lower body mass from 2 weeks onwards (Fig 1B). After 10 weeks of UA treatment, mice weighed 23.5% less than controls. Lower body weight in UA-treated mice resulted from the decreased fat mass (−61.3%) and a lower fat/body mass ratio (−70%) (Fig 1C). These pronounced changes in body composition were neither caused by a change in feeding behavior nor by a change in fat absorption in the intestine because food intake and fecal TG content were similar in the vehicle and UA-treated groups (Fig 1D and 1E). However, when UA was given to male C57BL/6 mice that were kept on a chow diet for a testing period of 6 weeks (S1A Fig), no significant differences in body weight, food intake, and fat mass were observed (S1B–S1D Fig).

Besides its preventive role in dietary obesity, to further assess whether UA could ameliorate genetic obesity, UA was given to male *ob*/*ob* mice, a widely used genetic model of obesity [31]. After 6 weeks of treatment, UA-treated *ob*/*ob* mice showed significantly lower body weight (−17.6%) than vehicle controls (S2A Fig). Despite similar food intake being shown between the 2 groups (S2B Fig), the fat tissue of UA-treated *ob*/*ob* mice weighed 16.9% less than that of vehicle controls (S2C Fig). Together, these results suggest that UA could efficiently prevent both HFD-induced and genetic obesity in mice.

Next, to further compare the preventive effect of UA on body weight control with an FDA-approved drug for obesity management, orlistat [32], 3 groups of male C57BL/6 mice kept on an HFD were treated with either 30 mg/kg/day of UA or 15 mg/kg/day of orlistat or vehicle for a testing period of 6 weeks. Compared to vehicle controls, both UA-treated mice and orlistat-treated mice showed significantly lower body weight after 2 weeks of treatment (S3A Fig). No significant difference in food intake was observed between the UA-treated group and the orlistat-treated group (S3B Fig), suggesting that the preventive effect of UA on HFD-induced body weight gain is similar to that of orlistat.

Given the safety of the compound, treated C57BL/6 mice tolerated UA well during the 10 weeks of compound administration with no apparent side effects. Plasma activities of the liver transaminases alanine aminotransferase (ALT) (−22%) and aspartate aminotransferase (AST) (−26.8%) were lower in UA-treated mice than that of controls (S4A and S4B Fig), arguing for normal or even improved liver function. Similarly, blood urea nitrogen (BUN) and creatinine (CREA) concentrations in plasma were similar in both groups (S4C and S4D Fig), providing evidence for normal kidney function. Together, these results show that UA treatment protects mice against obesity without obvious toxic signs within the tested dose and period.

### UA prevents HFD-induced insulin resistance and glucose intolerance in mice

Next, we assessed the effects of UA on glucose metabolism. Plasma glucose concentrations (−16.2% and −29.4% after 5 and 10 weeks of treatment, respectively) and insulin (−16.7% and −37.1% after 5 and 10 weeks of treatment, respectively) were lower in UA-treated mice than those in controls (Fig 1F and 1G). Based on levels of fasting glucose and insulin, the calculated homeostasis model assessment-estimated insulin resistance (HOMA-IR) index was lower after 5 weeks (−16.2%) and 10 weeks (−29.4%) in UA-treated mice than that of the vehicle group (Fig 1H). Insulin sensitivity and glucose tolerance were further evaluated using the insulin tolerance test (ITT) and glucose tolerance test (GTT), showing improved insulin sensitivity (Fig 1I and 1J) and glucose tolerance (Fig 1K and 1L) in UA-treated mice over controls. Finally, insulin sensitivity was assessed by hyperinsulinemic–euglycemic clamp assay. Higher levels of glucose infusion rate (GIR) were found in the UA-treated mice than controls during the clamp (Fig 1M). This increase in GIR was caused by elevated whole-body glucose uptake as well as the decreased endogenous hepatic glucose production during the clamp (Fig 1N and 1O). Consistently, UA-treated mice showed higher levels of insulin-stimulated glucose uptake in gastrocnemius muscle, BAT, and WAT than controls during the clamp (Fig 1P). Note that similar levels of glucose were observed between control and UA-treated mice under normal diet feeding (S1E Fig), consistent with their comparable body weight. Altogether, these results showed that UA protects mice from HFD-induced hyperglycemia and insulin resistance.

### UA prevents HFD-induced systemic inflammation and liver steatosis

Besides insulin resistance and glucose intolerance, systemic inflammation and fatty liver are 2 common metabolic manifestations that often accompany obesity [33]. To detect whether UA attenuates HFD-induced inflammation in mice, levels of plasma tumor necrosis factor-α (TNF-α) and interleukin 6 (IL-6) were measured. Lower concentrations of TNF-α and IL-6 were shown in UA-treated mice than in controls (S5A and S5B Fig). Moreover, histological evaluation of liver sections revealed that UA prevents HFD-induced fatty liver in mice (S5C Fig). Consistently, liver TG content (S5D Fig) and liver weight (S5E Fig) were 28.7% and 23.1% lower in UA-treated mice than in controls, respectively. Together, these results suggest that UA prevents HFD-induced systemic inflammation and liver steatosis.

### UA reverses HFD-induced obesity and dysfunctional glucose homeostasis in mice

To determine whether UA, in addition to its preventive action on obesity and hyperglycemia, also has therapeutic potential, we used obese mice that were kept on an HFD for 8 weeks prior to UA or vehicle treatment. UA treatment was initiated when the obese mice had a total body weight of approximately 35 g and continued for 10 weeks (S6A Fig). From 4 weeks onwards, UA-treated mice exhibited a significantly lower body weight than vehicle-treated mice (−6.4%) (S6B Fig). By 10 weeks, UA-treated mice weighed 23.8% less than control mice (S6B Fig). For UA-treated mice, the body weight slightly increased during the first 6 weeks of treatment, then returned to the initial body weight after 7 weeks, and finally decreased to a lower than initial body weight after 10 weeks (−4.2%) (S6B Fig). Changes in body weight exclusively resulted from decreased fat mass (−44.9%) and a decreased fat/body weight ratio (−39.2%) (S6C Fig) after 6 weeks of treatment. Lean mass was not affected by UA. Daily food intake was similar in both groups (S6D Fig).

After 6 weeks of treatment, plasma TG concentrations were 30.4% lower in UA-treated mice than in controls (S6E Fig), and levels of plasma glucose (−26%) (S6F Fig) and insulin (−21.6%) (S6G Fig), as well as the related HOMA-IR index (−42.4%) (S6H Fig), were lower in UA-treated mice than in controls. The glucose tolerance and insulin sensitivity were mildly but statistically improved in UA-treated mice, as shown by GTT and ITT (S6I–S6L Fig and S6M–S6P Fig, respectively). Together, these results showed that UA reverses HFD-induced obesity and dysfunctional glucose homeostasis in mice, possessing therapeutic potential for human obesity treatment.

### UA increases EE in mice

**Fig 2:**
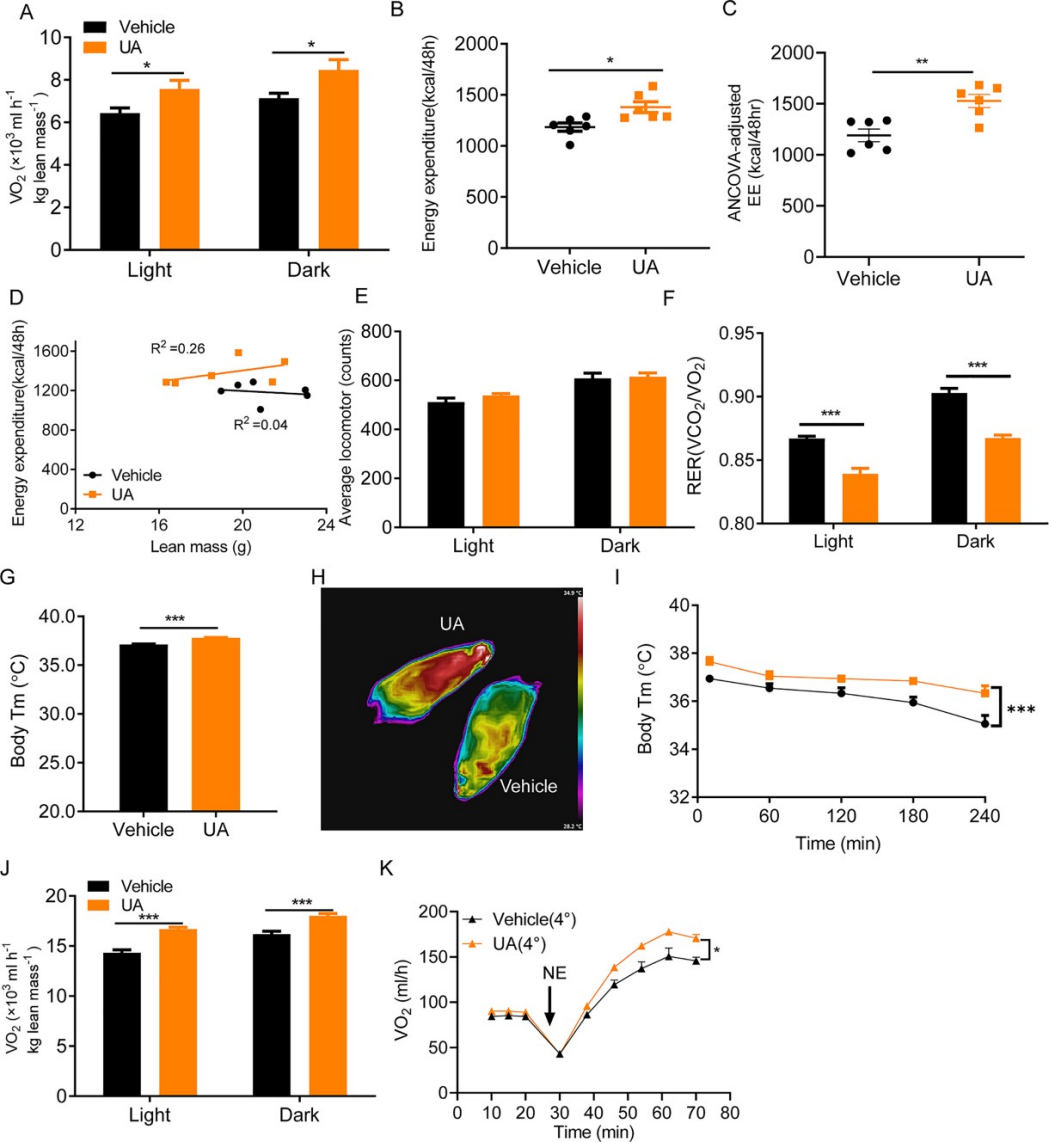
UA increases EE and adaptive thermogenesis. (A) Oxygen consumption measured by calorimetry in UA-treated and control mice (*n* = 6). (B) EE. (C) EE values adjusted for lean mass using ANCOVA. (D) Regression of EE against lean mass. (E) Locomotor activity measured by calorimetry in UA-treated and control mice (*n* = 6). (F) RER. (G) The body temperature of mice placed at room temperature (21°C) (*n* = 6). (H) Representative infrared images of mice. Images are displayed using the rainbow high-contrast color palette in FLIR Research. (I) Body temperature of mice placed at 4°C at the indicated time points (n = 6). (J) Oxygen consumption. (K) NE-stimulated whole-body oxygen consumption in mice at 4°C (*n* = 6). The underlying data for this figure can be found in S1 Data. EE, energy expenditure; NE, norepinephrine; RER, respiratory exchange ratio; UA, urolithin A.

To explore the cause of lower body mass in UA-treated mice, EE was measured by indirect calorimetry. We found significantly higher oxygen consumption (Fig 2A) as well as EE (Fig 2B–2D) in UA-treated mice than that of controls during both the light and dark phases, while locomotor activity was slightly higher but not statistically different between UA-treated and control mice during both light (*p* = 0.06) and dark phases (Fig 2E). The respiratory exchange ratio (RER), a parameter reflecting the relative use of carbohydrate versus lipid as source of energy, was markedly lower in UA-treated mice than in controls (Fig 2F), suggesting that UA could shift energy source towards lipid. We noticed that UA-treated mice exhibited about 0.7°C higher body temperature than that of controls at ambient temperature (Fig 2G). This temperature difference was also confirmed by an infrared camera (Fig 2H). In line with this, UA-treated mice maintained their body temperature better than controls (Fig 2I) when placed at 4°C. Higher oxygen consumption was shown both in the steady state (Fig 2J) as well as in the norepinephrine (NE)-stimulated non-steady state (Fig 2K) of UA-treated mice than that of controls at 4°C, indicating that UA was able to activate nonshivering thermogenesis upon cold stress. Collectively, these results show that UA enhances EE by promoting adipose tissue thermogenesis in mice, which may lead to the observed lower body weight in UA-treated mice. However, the opposite may also be true: UA treatment could cause a lean phenotype first, which could subsequently lead to increased thermogenesis because in general, a lean phenotype is associated with increased thermogenic adipocyte function. To test it, thermogenesis was measured in mice received UA or vehicle for 7 days (S7 Fig), when the body weight was similar between 2 groups of mice (control, 21.85 ± 0.54 g versus UA, 21.82 ± 0.85 g). A significantly higher oxygen consumption was shown in UA-treated mice than in controls (S7 Fig), suggesting that functional thermogenesis happens earlier than body weight changes.

### UA promotes BAT thermogenesis and induces inguinal white fat browning in an adipocyte-autonomous manner

**Fig 3:**
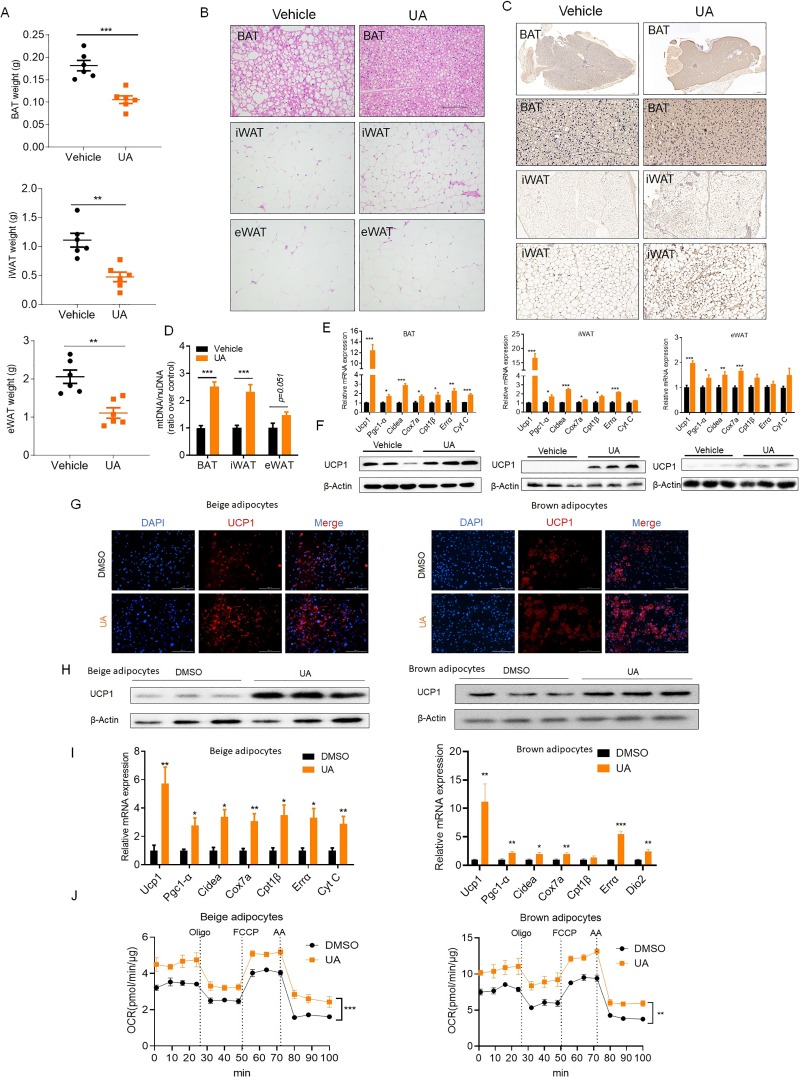
UA promotes thermogenesis in BAT and induces browning of iWAT. (A) Tissue weights of BAT, iWAT, and eWAT in UA-treated mice and controls for a period of 10 weeks under HFD feeding (*n* = 6). (B) Morphological changes in the 3 depots of fat shown by HE staining. (C) Immunohistochemistry staining of UCP-1 in sections of 3 fat depots. (D) mtDNA copy number in BAT, iWAT, and eWAT (*n* = 6). (E) mRNA levels of thermogenic genes in BAT, iWAT, and eWAT (*n* = 6). (F) Immunoblot analysis of UCP-1 in BAT, iWAT, and eWAT. (G) Immunofluorescence and (H) immunoblot analysis of UCP-1 in primary cultured adipocytes from iWAT and BAT. (I) mRNA levels of thermogenic genes in primary cultured adipocytes from iWAT and BAT of UA-treated mice and controls (*n* = 6). (J) OCR measured by a Seahorse analyzer in brown adipocytes and beige adipocytes treated with or without UA. The underlying data for this figure can be found in S1 Data. AA, antimycin A; BAT, brown adipose tissue; Cidea, Cell death inducing DFFA like effector a; Cox7a, Cytochrome c oxidase subunit 7a1; Cpt1b, Carnitine palmitoyltransferase 1B; Cyt c, Cytochrome c; Dio2, Deiodinase 2; Errα, Estrogen related receptor α; eWAT, epididymal WAT; FCCP, carbonyl cyanide-4-(trifluoromethoxy) phenylhydrazone; HE, hematoxylin–eosin; HFD, high-fat diet; iWAT, inguinal WAT; mtDNA, mitochondrial DNA; nuDNA, nuclear DNA; OCR, oxygen consumption rate; Pgc1-α, Peroxisome proliferator-activated receptor gamma coactivator 1α; UA, urolithin A; UCP-1, Uncoupling protein 1; WAT, white adipose tissue.

To further study the thermogenic effect of UA, tissue weights of BAT, inguinal (iWAT), and epididymal WAT (eWAT) were measured. All 3 adipose depots weighed less (BAT, −37.4%; iWAT, −57.2%; eWAT, −46.1%) in UA-treated mice than in controls (Fig 3A). Histological analysis revealed smaller adipocyte sizes in BAT, iWAT, and eWAT of UA-treated mice compared with controls (Fig 3B and S8 Fig), suggesting that UA treatment causes resistance to HFD-induced adipose tissue hypertrophy. Immunohistochemistry staining of Uncoupling protein 1 (UCP-1) was higher in BAT and iWAT of UA-treated mice than of controls (Fig 3C). BAT (2.5-fold), iWAT (2.1-fold), and eWAT (1.4-fold) exhibited an increased amount of mitochondrial DNA (mtDNA) (Fig 3D). Furthermore, mRNA expression levels of mitochondrial thermogenic genes were significantly higher in the 3 fat depots of UA-treated mice than those of controls (Fig 3E). Finally, BAT and iWAT of UA-treated mice had higher protein levels of UCP-1 than untreated controls (Fig 3F). Taken together, these data indicate that the low weight gain in UA-treated mice is due to the increased mitochondrial biogenesis and activated thermogenic program in BAT and iWAT.

To further assess whether UA increases the “browning propensity” and BAT activation in an adipocyte-autonomous manner, preadipocytes from inguinal fat and interscapular brown fat were isolated and differentiated in the presence or absence of UA. After differentiation, higher levels of UCP-1 protein (shown by immunostaining and western blotting) (Fig 3G and 3H) and elevated mRNA levels of typical browning markers were observed in UA-treated adipocytes than those of controls (Fig 3I). Adipocyte thermogenic capacity is mitochondrial uncoupling, as determined by respiration. Therefore, mitochondrial oxygen consumption rate (OCR) was measured by a Seahorse analyzer. The results showed that the basal, uncoupled, and maximal OCRs were significantly higher in UA-treated brown adipocytes, as well as white adipocytes, than those of controls (Fig 3J). Together, these results demonstrate that UA promotes browning of inguinal white fat and activates BAT in a cell-autonomous manner.

### The β-adrenergic receptor is not a major contributor for UA-stimulated thermogenesis

**Fig 4:**
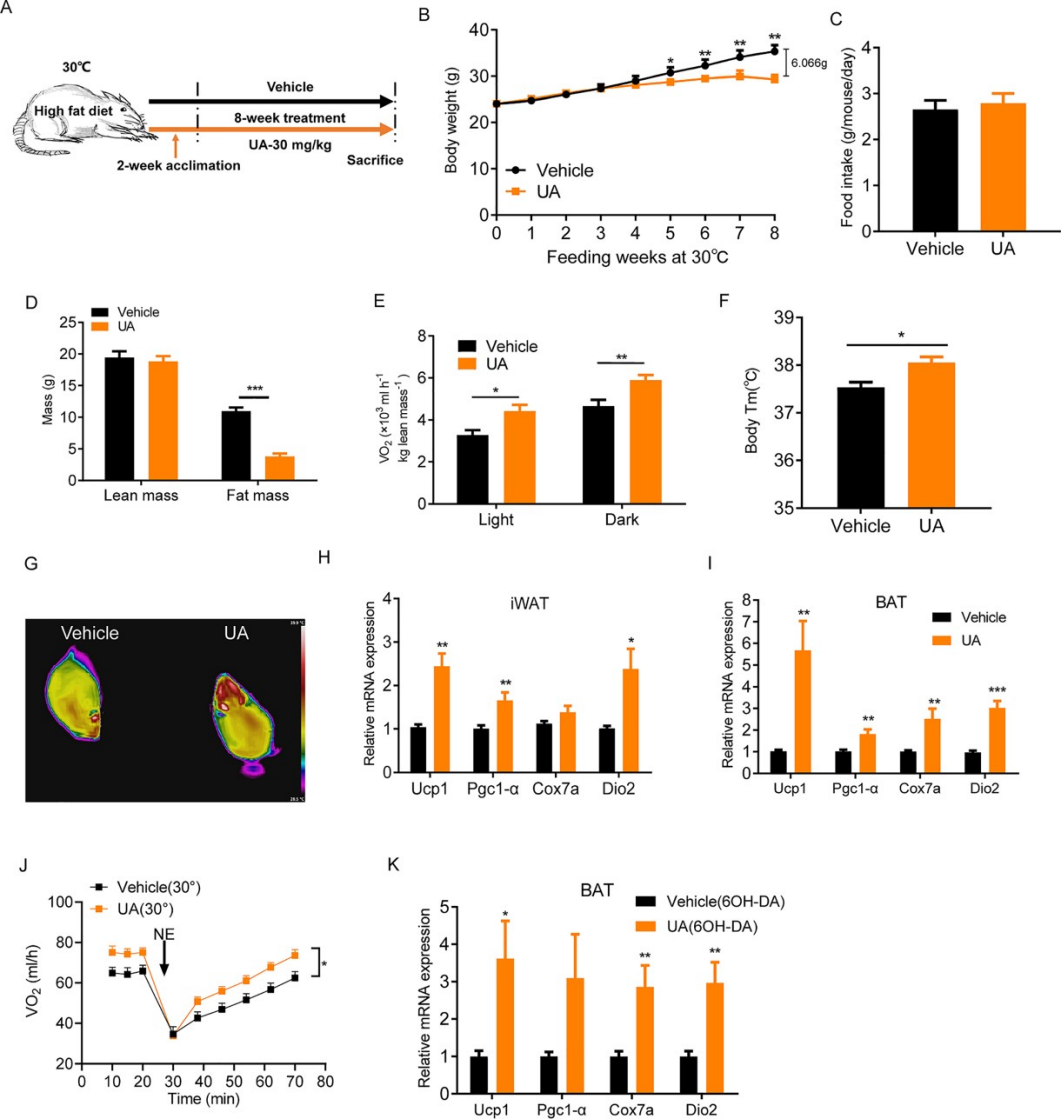
UA-mediated thermogenesis and body weight control in mice is preserved at thermoneutrality. (A) Schematic diagram of mice treatment. (B) Body weight of HFD-fed mice treated with UA or vehicle at 30°C (*n* = 6). (C) Daily food intake of mice at 30°C (*n* = 6). (D) Body composition measured by DEXA scan after 6 weeks of treatment. (E) Oxygen consumption of mice tested for 24 h (*n* = 6). (F) The body temperature of mice at 30°C (*n* = 6). (G) Representative infrared images of the vehicle and UA-treated mice. mRNA levels of thermogenic genes in (H) iWAT and (I) BAT from UA-treated or vehicle control mice at 30°C (*n* = 6). (J) NE-stimulated whole-body oxygen consumption in UA-treated mice and controls mice at 30°C. (K) mRNA levels of thermogenic genes in denervated BAT of UA-treated or vehicle mice at room temperature. The underlying data for this figure can be found in S1 Data. BAT, brown adipose tissue; Cox7a, Cytochrome c oxidase subunit 7a1; DEXA, dual-energy X-ray absorptiometry; Dio2, Deiodinase 2; HFD, high-fat diet; iWAT, inguinal white adipose tissue; NE, norepinephrine; Pgc1-α, Peroxisome proliferator-activated receptor gamma coactivator 1α; UA, urolithin A; Ucp1, Uncoupling protein 1.

One of the well-established mechanisms of nonshivering thermogenesis involves activation of the β-adrenergic receptors (β-ARs) [34–36]. We therefore tested whether β-ARs mediate UA-induced activation of BAT and browning of WAT. Mice were placed in a thermoneutral environment (30°C) (Fig 4A) where stimulation of β-ARs is blunted and nonshivering thermogenesis is not required [37]. At thermoneutrality, UA-treated mice (8 weeks) exhibited lower body weight (−17.2%) than controls (Fig 4B), despite this difference in body weight being smaller (6.066 g in thermoneutrality versus 7.865 g at room temperature) than that observed at room temperature. Calorie intake was similar between the 2 groups of mice studied (Fig 4C). The fat mass was lower in UA-treated mice than that of controls (Fig 4D). This could contribute to the lower body weight of UA-treated mice. In accordance with the results acquired at room temperature, oxygen consumption was 35% and 26% higher in the UA group than in controls during the light and dark cycles, respectively (Fig 4E). UA-treated mice had a higher body temperature (+0.52°C) than that of controls at thermoneutrality (Fig 4F and 4G). Similar to mice kept at ambient temperature, mRNA levels of thermogenic genes were higher in the iWAT and BAT of UA-treated mice than in controls at thermoneutrality (Fig 4H and 4I). Furthermore, when NE was injected into mice at thermoneutrality, significantly higher O_2_ consumption was shown in UA-treated mice than in controls (Fig 4J), suggesting β-ARs are not necessary for the action of UA. To further confirm this, we employed a chemical approach to denervate the sympathetic nervous system (SNS) of BAT by microinjection of a specific neurotoxin 6-hydroxydopamine (6-OHDA) into brown fat of mice. The result showed that mRNA expressions of thermogenic markers were higher in BAT of UA-treated mice than that of controls (Fig 4K). Together, these results showed that β-AR activation is not necessary for UA-stimulated thermogenesis.

### Adipose tissue TH signaling is involved in the UA-stimulated nonshivering thermogenesis

**Fig 5:**
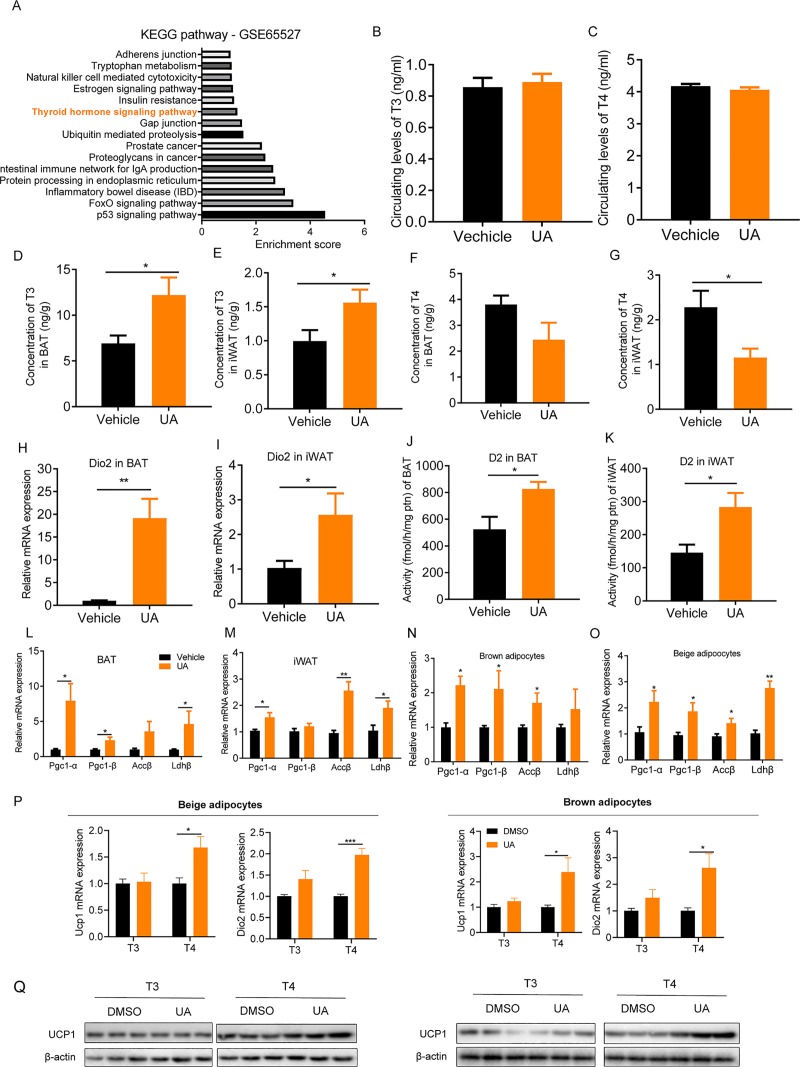
Levels of TH and its responsive gene expression. (A) KEGG pathway enrichment analysis in the publicly available GEO data set. Circulating levels of (B) T3 and (C) T4 in HFD-fed mice with UA or vehicle treatment. The concentration of T3 in (D) BAT and (E) iWAT of HFD-fed mice with UA or vehicle treatment. The concentration of T4 in (F) BAT and (G) iWAT of HFD-fed mice with UA or vehicle treatment. (H) mRNA expression of Dio2 in BAT and (I) iWAT of HFD-fed mice with UA or vehicle treatment. The activity of D2 in (J) BAT and (K) iWAT of HFD-fed mice with UA or vehicle treatment. mRNA expression of thyroid-hormone-responsive genes in (L) BAT and (M) iWAT of HFD-fed mice with UA or vehicle treatment. *n* = 6 for each experiment. mRNA expression of TH-responsive genes in primary cultured (N) white adipocytes and (O) brown adipocytes in the presence or absence of UA (*n* = 6). (P) mRNA expressions of UCP1 and Dio2 in beige adipocytes and brown adipocytes. (Q) Immunoblot analysis of UCP-1 protein in beige adipocytes and brown adipocytes. The underlying data for this figure can be found in S1 Data. Accβ, Acetyl-CoA carboxylase beta; BAT, brown adipose tissue; Dio2, Deiodinase 2; FoxO, The forkhead box O; GEO, Gene Expression Omnibus; GSE, GEO Series; HFD, high-fat diet; IBD, inflammatory bowel disease; IgA, immunoglobulin A; iWAT, inguinal white adipose tissue; KEGG, Kyoto Encyclopedia of Genes and Genomes; Ldhβ, Lactate dehydrogenase B; Pgc1-α, Peroxisome proliferator-activated receptor gamma coactivator 1α; TH, thyroid hormone; T3, triiodothyronine; T4, tetraiodothyronine; UA, urolithin A; UCP-1, Uncoupling protein 1.

To find any clues to the potential underlying mechanism implicated in UA-mediated thermogenesis, we analyzed the available gene expression in UA-treated prostate cancer cells (LNCaP) from the Gene Expression Omnibus (GEO) data set (GSE65527). Kyoto Encyclopedia of Genes and Genomes (KEGG) pathway analysis of the data set showed that the TH signaling pathway, which plays key roles in the regulation of thermogenesis and body temperature in humans and animals [38–40], is highly up-regulated after UA treatment (Fig 5A). To test whether TH signaling is involved in UA-mediated thermogenesis in mice, the circulating levels of triiodothyronine (T3) and tetraiodothyronine (T4) were assayed. There was no significant difference in levels of circulating T4 or T3 between UA-treated mice and vehicle controls (Fig 5B and 5C). However, the activated form of TH is T3, which is mainly converted from inactive T4 in its target tissue [41]. Adipose tissue is one of its target tissues [42]. When the 2 hormones were measured in adipose tissue, higher concentrations of T3 were detected in the BAT (1.76-fold) and iWAT (1.56-fold) of the UA-treated mice than in controls (Fig 5D and 5E); a similar pattern of T3 was also observed in *ob*/*ob* mice (S2D Fig), whereas levels of T4 tended to decrease in the UA-treated group (Fig 5F and 5G), suggesting that UA treatment enhances the production of active T3 in adipose tissue, probably from converting the inactive T4. Consistent with this, we noticed higher mRNA levels of Deiodinase 2 (Dio2) (iWAT, 2.49-fold and BAT, 19.1-fold), a gene encoding the rate-limiting enzyme D2 that is responsible for the conversion of T4 to T3, and increased enzyme activities of D2 (iWAT, 1.94-fold and BAT, 1.57-fold) in the UA group than in controls (Fig 5H–5K); a similar pattern of Dio2 mRNA expression was also observed in *ob*/*ob* mice (S2E Fig). Expression levels of TH-responsive genes (Peroxisome proliferator-activated receptor gamma coactivator 1α [Pgc1α], Pgc1β, Acetyl-CoA carboxylase beta [Accβ], and Lactate dehydrogenase B [Ldhβ]) were also significantly higher in the BAT and iWAT of UA-treated mice than in control mice (Fig 5L and 5M). Consistently, in primary cultured white adipocytes and brown adipocytes, significantly higher expression levels of TH-responsive genes were shown in UA-treated cells than those of controls (Fig 5N and 5O). Together, these results indicate that TH is involved in the UA-mediated thermogenesis in mice.

To further explore TH signaling in the UA-mediated thermogenesis in vitro, primary cultured white adipocytes and brown adipocytes were differentiated to adipocytes for 6 days in the standard differentiation medium for beige or brown adipocytes without UA treatment. Cells were then incubated with either 100 nM T3 or 100 nM T4 in the presence or absence of UA. Higher levels of UCP-1 and DIO2 expression were observed in UA -treated cells incubated with T4 than in control cells (Fig 5P and 5Q). These results suggest that UA converts inactive T4 to activate T3, which induces thermogenesis in vitro.

### The beneficial metabolic effect of UA depends on TH

**Fig 6:**
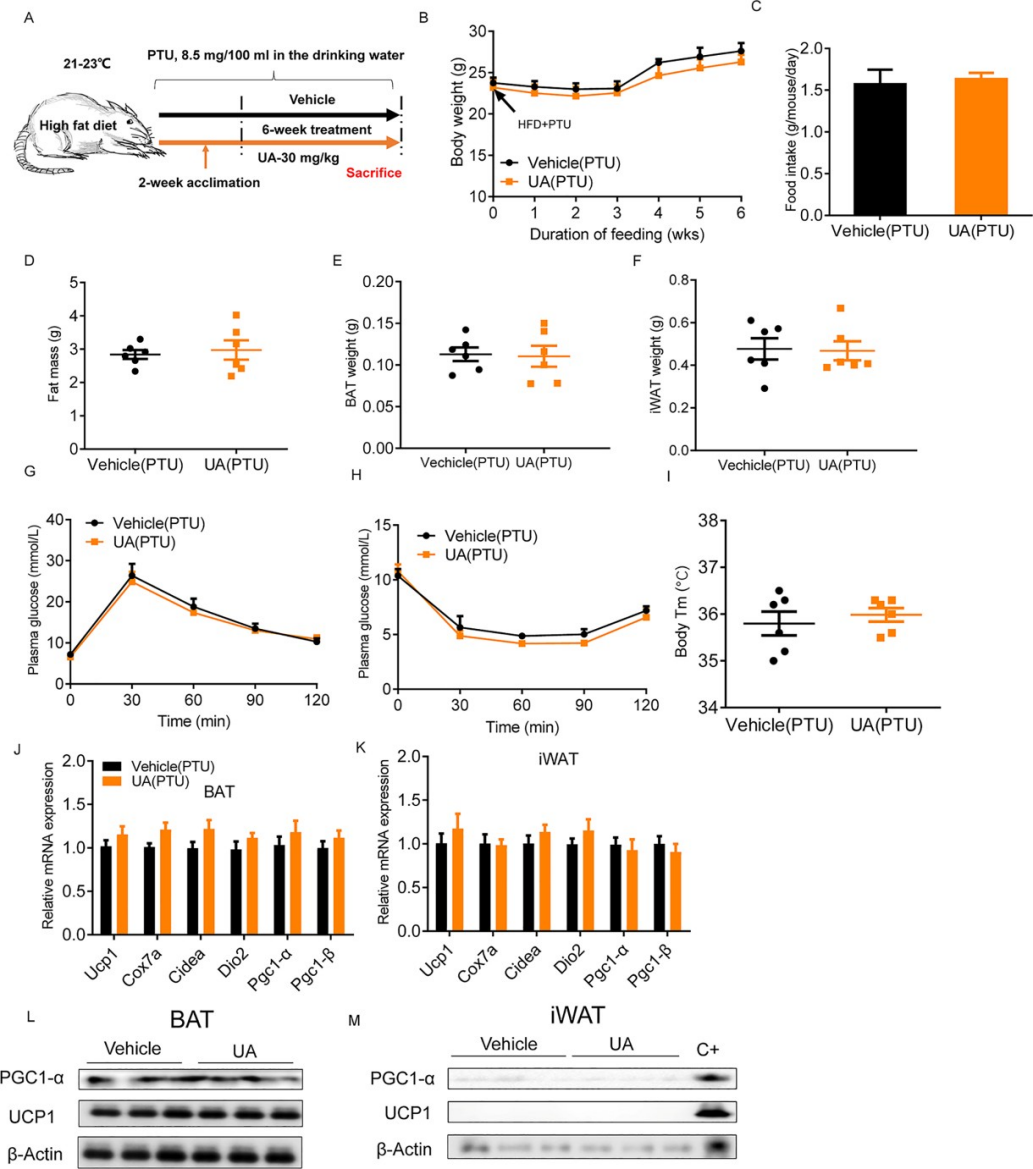
Blocking the production of TH abrogates the beneficial effects of UA on thermogenesis and body weight control. (A) Schematic diagram of mice treatment. UA- and vehicle-treated mice were given PTU (8.5 mg/100 ml in the drinking water), an inhibitor of TH synthesis, for a period of 7-week HFD feeding. (B) Body weight time course (*n* = 6). (C) Daily food intake (*n* = 6). Weight of (D) total fat mass, (E) BAT, and (F) iWAT (*n* = 6). (G) GTT and (H) ITT performed in mice (*n* = 6). (I) Body temperature in mice placed at 21°C (*n* = 6). mRNA expression of thermogenic genes in (J) BAT and (K) iWAT (*n* = 6). Immunoblot of UCP-1 and PGC-1α protein in (L) BAT and (M) iWAT (*n* = 3). C+: positive control from BAT. The underlying data for this figure can be found in S1 Data. BAT, brown adipose tissue; Cidea, Cell death inducing DFFA like effector a; Cox7a1, Cytochrome c oxidase subunit 7a1; Dio2, Deiodinase 2; GTT, glucose tolerance test; HFD, high-fat diet; ITT, insulin tolerance test; iWAT, inguinal white adipose tissue; Pgc1-α, Peroxisome proliferator-activated receptor gamma coactivator 1α; PTU, propylthiouracil; TH, thyroid hormone; UA, urolithin A; UCP-1, Uncoupling protein 1.

To corroborate the role of TH in UA-mediated thermogenesis and the prevention of diet-induced weight gain in vivo, a hypothyroidism mouse model was created (S9 Fig) by treating the mice with propylthiouracil (PTU), an inhibitor of TH production [43], for 6 weeks (Fig 6A). PTU treatment completely blunted the preventive effect of UA on body weight gain (Fig 6B). Food intake was similar in both groups (Fig 6C). Total fat, BAT, and iWAT weights were similar between UA-treated and control groups after PTU treatment (Fig 6D–6F). The beneficial effect of UA on glucose homeostasis also disappeared after PTU treatment, showing similar levels of glucose and courses of GTT and ITT (Fig 6G and 6H). Comparable body temperatures in UA-treated and control mice (Fig 6I) suggested that the thermogenic effect of UA is lost upon PTU administration. Consistent with this observation, the mRNA expression levels of thermogenic genes in iWAT and BAT were not statistically different in UA-treated mice and vehicle controls after PTU administration (Fig 6J and 6K). A similar amount of Pgc1α and UCP-1 protein were shown in the BAT of both groups after PTU treatment (Fig 6L). In iWAT of PTU-treated mice, Pgc1α and UCP-1 protein were undetectable in both groups of mice (Fig 6M). Together, these results showed that inhibition of TH production abolishes the beneficial metabolic effect of UA in mice.

**Fig 7:**
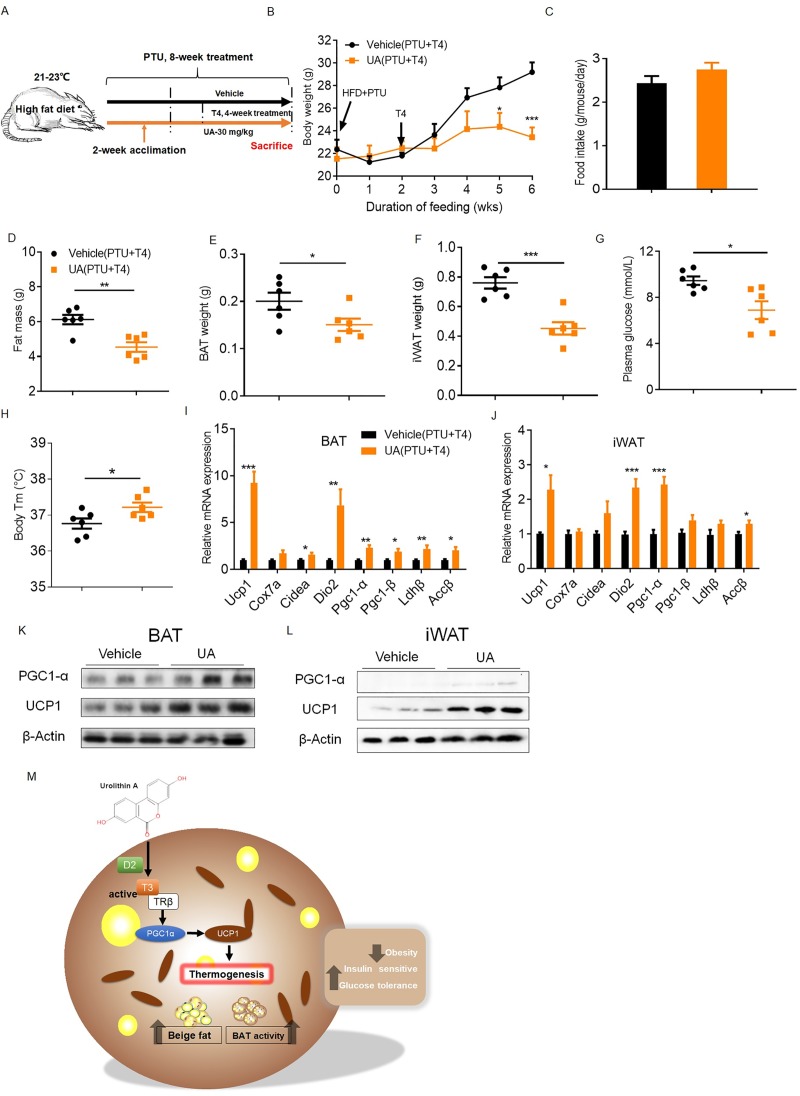
Exogenous T4 administration restores the actions of UA in PTU-treated mice. (A) Schematic diagram of mice treatment. UA-treated and control mice were initially treated with PTU (8.5 mg/100 ml in the drinking water) for 2 weeks and subsequently received PTU plus T4 (1 μg/100 g in 0.2% BSA-PBS) for a period of 4 weeks. (B) Body weight time course. (C) Daily food intake. Weight of (D) total fat mass, (E) BAT, and (F) iWAT (*n* = 6). (G) Levels of plasma glucose. (H) Body temperature of mice placed at 21°(C). mRNA expression of thermogenic genes in (I) BAT and (J) iWAT. Immunoblot of UCP-1 and PGC-1α protein in (K) BAT and (L) iWAT (*n* = 3). (M) Schematic diagram of actions of UA in the regulation of thermogenesis through TH. The underlying data for this figure can be found in S1 Data. Accβ, Acetyl-CoA carboxylase beta; BAT, brown adipose tissue; BSA, bovine serum albumin; Cidea, Cell death inducing DFFA like effector A; Cox7a, Cytochrome c oxidase subunit 7a1; Dio2, Deiodinase 2; HFD, high-fat diet; iWAT, inguinal white adipose tissue; Ldhβ, Lactate dehydrogenase B; Pgc1-α, Peroxisome proliferator-activated receptor gamma coactivator 1α; PTU, propylthiouracil; TRβ, TH Receptor β; T3, triiodothyronine; T4, tetraiodothyronine; UA, urolithin A; UCP-1, Uncoupling protein 1.

Because UA treatment increases the expression and activity of D2 (Fig 5H–5K), we reasoned that the main function of UA in TH production is to convert inactive T4 to active T3, which subsequently activated thermogenesis. To test this, exogenous inactive T4 instead of active T3 was given to PTU-treated mice. UA-treated and control mice were initially treated with PTU for 2 weeks and subsequently received PTU plus T4 (1 μg/100 g/day) for a period of 4 weeks (Fig 7A). Already, after 3 weeks of T4 treatment, body weights of UA-treated mice were 18.33% lower than those of controls (Fig 7B) despite similar food intake (Fig 7C). In line with lower body weight, the total fat mass and BAT and iWAT mass decreased in UA-treated mice by 25.8%, 24.9%, and 40%, respectively (Fig 7D–7F). Levels of plasma glucose were 25.2% lower in UA-treated mice than in controls after 3 weeks of T4 supplementation (Fig 7G). Increased body temperature by 0.8°C (Fig 7H) was accompanied by elevated transcript levels of thermogenic genes implicated in the activation of BAT and browning of iWAT of UA-treated versus control mice after 4 weeks of T4 supplementation (Fig 7I and 7J). Finally, T4 treatment resulted in higher UCP-1 and PGC-1α protein levels in BAT and iWAT of UA-treated than in control mice (Fig 7K and 7L). Together, these results demonstrate that UA-mediated thermogenesis depends on TH. Inhibition of TH production prevents, whereas exogenous T4 supply restores, the beneficial effects of UA on body mass control and glucose homeostasis.

## Discussion

The prevalence of obesity has nearly tripled worldwide during the past 30 years [44–47]. Tremendous efforts have been made to develop effective antiobesity strategies. Unfortunately, strategies based on diet, exercise, and behavioral adaptation modifications have turned out to be insufficient for the long-term prevention of obesity in the majority of the population [48–51], and pharmacotherapy of obesity has been repeatedly found to be unsafe [52]. Therefore, novel, effective, and safe antiobesity interventions are urgently needed. In this study, we showed that UA, a gut-microflora–derived metabolite of natural products, prevents and also reverses HFD-induced obesity by promoting thermogenesis in BAT and iWAT. Besides the antiobesity effect, UA causes a series of beneficial metabolic effects, including improved insulin sensitivity and reduced systemic inflammation and fatty liver, which are at least partially due to weight loss. The mechanism of UA-stimulated thermogenesis is summarized in Fig 7M, showing that UA enhances adipose tissue production of T3, which subsequently activates transcription and translation of the thermogenic genes PGC1a and UCP-1, leading to higher EE and lower body weight.

In regard to the effect of UA on body weight control, we showed that the lower body weight (lower fat mass but similar lean mass) is due neither to the food intake nor to intestine fat absorption. This raises the question of how UA treatment decreases fat accumulation. In principle, several factors may contribute to it: i) decreased adipose tissue lipid synthesis. Indeed, it is reported that UA decreases adipogenic protein and gene expression in primary cultured human adipocytes and murine 3T3-L1 adipocytes, leading to less TG accumulation [29,30]; ii) increased lipid catabolism and subsequent utilization; and iii) decreased lipid uptake by the intestine. Our data support that lower lipid synthesis and higher lipid utilization together contribute to UA-mediated decreased fat accumulation in mice. The decreased lipid synthesis is demonstrated by lower mRNA and protein levels of TG synthesis markers in the iWAT of UA-treated mice than in controls (S10 Fig), as well as in primary cultured white and brown adipocytes (S11 Fig). The increased lipid utilization is demonstrated in UA-treated mice by i) a lower RER, reflecting that lipids instead of carbohydrates are used as the main energy substrate; and ii) more EE is directly shown by pronounced higher levels of heat production and elevated core body temperature in UA-treated mice than in controls, which could have a profound contribution to energy consumption and therefore was explored further in this study.

Thermogenesis occurs in several tissues, including BAT, WAT, and skeletal muscle [53]. Here, several pieces of evidence collectively support that UA treatment enhances heat production by increasing the activity of BAT and inducing browning of iWAT: i) morphologically, BAT and iWAT of UA-treated mice resisted HFD-induced hypertrophy, maintaining a relatively normal appearance; ii) by immunohistochemistry, positive staining of UCP-1, a typical marker of BAT and browning of WAT, is higher in BAT and iWAT of UA-treated mice than in controls; iii) more mitochondria were shown by mtDNA copy number in BAT and iWAT of UA-treated mice; iv) mRNA levels of thermogenic markers and of UCP-1 protein were higher in the BAT and iWAT of UA-treated mice; and v) finally, infrared thermography revealed that UA-treated mice had a significantly higher interscapular BAT temperature than controls at room temperature. Together, these results show that UA directly enhances BAT thermogenesis and induces browning of iWAT, both of which contribute to higher levels of EE.

It is well established that thermogenesis in BAT and iWAT is regulated by the sympathetic nervous system through NE-mediated β-ARs and TH [54–59]. Our data rule out the involvement of β-ARs in the UA-mediated thermogenesis based on i) at thermoneutrality, at which stimulation of β-ARs is blunted, the effects of UA on body weight control and thermogenesis remained; and ii) when SNS of BAT was denervated, the effect of UA on tissue levels of DIO2 was maintained. Although there exist other thermogenic mechanisms, such as the proteasome system (S12A Fig), calcium (S12B Fig) [60], creatine cycling (S12C Fig) [61], and the glycolytic pathway (S12D–S12F Fig) [62], results derived from preliminary experiments in our study do not support a direct contribution of these pathways in the UA-mediated thermogenesis (S12 Fig). Instead, our data emphasize the importance of TH in the UA-mediated thermogenesis in BAT and iWAT. First, the mRNA expression and activities of the enzyme D2, which is responsible for the conversion of T4 to T3, were higher in BAT and iWAT of UA-treated mice than in controls, with a consequently higher concentration of T3 in the corresponding tissues. Consistently, in the presence of inactivated T4, higher levels of Dio2 and Ucp1 were shown in UA-treated primary cultured white and brown adipocytes than in controls (Fig 5P and 5Q). More directly, blocking the production of TH in mice abrogated the effects of UA on body weight control and nonshivering thermogenesis in BAT and iWAT. Conversely, restoration of TH production rescued the beneficial effects of UA. Together, these evidences strongly support that TH is indispensable for the beneficial effects of UA in mice.

It is worth noting that mice only respond to UA under an HFD, but not under a chow diet. This diet-dependent antiobesity effect is widely present in compound-treated normal mice [63,64], as well as in genetically modified mouse models [65]. The underlying mechanisms of these phenomena might be different and need further investigation. Mechanistically, the potential mechanism underlying the different effect of UA on mice body weight under an HFD and a chow diet might be due to tissue levels of T3, which is relatively low and may not reach concentrations at which it could significantly activate the thermogenic program in BAT and iWAT under normal diet feeding. Instead, higher levels of T3 were shown in UA-treated HFD mice than in normal-diet–feeding mice (Fig 5D and 5E (S12D–S12F Fig) [62], results derived from prelimin HFD increases tissue levels of T3 [66]. In fact, several similar lines of dose-dependent mechanism were reported on obesity research; for example, Growth differentiation factor 15 (GDF15) is widely accepted as a secreted protein that reduces food intake in mice [67]. Studies showed that the effect of GDF15 on food intake is dose-dependent [68]. More robust dose-dependent thermogenic evidence was derived from succinate study in mice, showing that 1.5% but not 1% of succinate could efficiently activate BAT thermogenesis, resulting in lower body weight [69]. Therefore, it is tempting to speculate that tissue levels of T3 may contribute to the different effects of UA under HFDs and normal diet feeding.

Safety is the top consideration for evaluating the potential clinical applications of antiobesity agents [70–74]. UA-mediated elevation of T3 concentrations in BAT and iWAT provokes us to consider the safety of UA application. Our data showed no obvious side effects and even an improvement for HFD-induced liver damage within the tested dose (30 mg/kg/day) and period of time (10 weeks). In rats, up to 5% of UA in the diet showed no adverse or toxic effects over a period of 90 days [75]. In addition, 50 mg/kg/day of UA treatment in mice for 8 months showed no adverse effects but an improvement of muscle grip strength and spontaneous exercise [27]. More importantly, the safety evaluation of UA in humans showed no side effects but improved mitochondrial function of skeletal muscle within the tested dose (a maximum of 1,000 mg/day) and period (56 days) [28,76,77]. In our study, we also measured the effect of UA on muscle functions in mice. A greater exercise capacity (grip experience: 1.86-fold, grip strength: 1.57-fold, and swimming time: 1.72-fold) in UA-treated mice than in controls was shown in S13 Fig. Together, results derived from both humans and rodents consistently proved no toxicity but a beneficial effect of UA on skeletal muscle functions. Also, a recent publication showed that UA significantly enhances gut barrier function and attenuates inflammatory bowel disease in preclinical models [20]. Thus, based on these findings, it is speculated that UA does not have the side effects such as gastrointestinal disorders [78] and myopathy [79] caused by orlistat. Despite the effects of UA on body mass and lipid metabolism not being reported in the human study [28], the potential clinical application of UA seems promising based on its current safety assessment as well as the beneficial effects in humans and mice.

In summary, the data presented in this study showed that UA has beneficial effects on body weight control and glucose homeostasis without causing any adverse effects in mice. Our work establishes a key role of UA in the regulation of lipid metabolism and offers great promise for identifying new therapies to combat obesity and its related metabolic disorders.

## Materials and methods

### Ethical approval

All animal studies were approved by and performed according to the guidelines of the ethics committee of the Northwest A&F University (Yangling, Shaanxi, China) (approval number: 20171208–010).

### Animals

Six-week–old male C57BL/6 mice purchased from the animal center of Xi’An Jiao Tong University (Xi’an, China) and 4-week-old male leptin-deficient *ob*/*ob* mice purchased from Tengxin Biotechnology Co. Ltd. (Chong Qing, China) were acclimated for 2 weeks in the animal facility of Northwest A&F University. Unless otherwise specified, C57BL/6 mice were raised under an HFD (composed of 26.2% protein, 34.9% fat, D12492; Xie Tong Corp., Nanjing, China), and *ob*/*ob* mice were raised under normal diet (1010009, Xie Tong Corp.). At the age of 8 weeks, C57BL/6 mice were started on receiving UA (30 mg kg^−1^ d^−1^, Jinan Feiteng Pharmaceutical Technology Co., Jinan, China) or orlistat (15 mg kg^−1^ d^−1^, Cayman Chemical Co., Ann Arbor, MI, USA) or vehicle (0.1% Tween 80) by gavage. At the age of 6 weeks, *ob*/*ob* mice were started on receiving UA or vehicle. All mice were raised with free access to food and water in a 12-hour light/dark animal facility. For BAT denervation, 6-OHDA was injected at 4 different sites of each BAT under anesthesia (1.5 μL per site of 10 mg/mL dissolved in 1% ascorbic acid). The mice were allowed to recover for a minimum of 10 days before further experiments. For inhibition of TH production, UA- and vehicle-treated mice were orally given PTU (8.5 mg/100 ml in the drinking water, Chengdu Jia Ye Biotechnology Co., Ltd., # 51-52-5; Nanjing, China), an inhibitor of TH synthesis, for a period of 6-week HFD feeding. For TH restoration experiments, UA-treated and control mice were initially treated with PTU for 2 weeks and subsequently received PTU plus T4 (1 μg/100 g in 0.2% bovine serum albumin [BSA]-PBS, daily IP injection, Shanghai Baoman Biotechnology Co., Shanghai, China) for a period of 4 weeks.

### GTTs and ITTs

For the GTT, overnight-fasted (approximately 14 h) mice received d-glucose (2 g/kg body weight) by IP injection. For ITT, 5-h fasted mice (from 6 AM to 11 AM) received recombinant human insulin at the concentration of 0.75 units/kg body weight. Levels of glucose were measured at indicated time points (0, 30, 60, 90, and 120 min) after injection.

### Hyperinsulinemic–euglycemic clamps assay

Hyperinsulinemic–euglycemic clamps were performed as previously described [80]. Briefly, an indwelling silastic catheter was implanted in the right jugular vein of anesthetized mice 7 d before the experiments. Mice with less than 10% weight loss were used for the subsequent studies. After overnight fasting (16 hours), conscious mice were infused with [3-^3^H]-glucose (HPLC purified; Perkin-Elmer Life Sciences, Waltham, MA, USA) at a rate of 0.05 μCi/min for 120 min for basal glucose turnover measurement. Then, clamps were performed for 140 min with a 3-min primed infusion of insulin (6.0 mU/[kg min]), followed by a continuous infusion of insulin (2.5 mU/[kg min]) and [3-^3^H]-glucose (0.1 μCi/min) and a variable infusion of 20% dextrose to maintain euglycemia (approximately 120 mg/dL). A bolus of 2-deoxy-D-[1-^14^C] glucose (10 μCi) was administrated to assess insulin-stimulated glucose uptake at *t* = 85 min. At the end of the clamps, mice were killed, and WAT, BAT, and gastrocnemius muscles were taken for subsequent analyses.

### Body composition

Lean and fat masses were evaluated using DEXA as described [81].

### Body temperature

The body temperature was recorded by a rectal probe (BAT-12, Physitemp, Clifton, NJ, USA).

### Calorimetry

This was determined using Oxymax/CLAMS as described [81]. Briefly, mice were acclimated to the metabolic chamber for 48 h before the formal experiment. Oxygen consumption (VO_2_), EE, and RER were measured with an Oxymax/CLAMS (Columbus Instruments, Columbus, OH, USA). An ANCOVA correction of EE with body composition as covariants was performed. For the evaluation of the effect of UA on whole-body thermogenic adipocyte activity, oxygen consumption was also measured acutely for mice receiving 1 mg/kg NE (Sigma-Aldrich, St. Louis, MO, USA) stimulation at 4°C and thermoneutrality (30°C) as described [82].

### Cellular respiration detection

Cellular OCRs were determined using a Seahorse XFe96 Extracellular Flux Analyzer (Seahorse Bioscience, Billerica, MA, USA). Cells from iWAT and BAT were seeded at 20,000 cells/well. Differentiation was induced as described above [69,83], and then the cells were analyzed. Before the measurements, cells were washed twice with assay medium and incubated in 175 μL of assay medium for 1 hour in an incubator without CO_2_ at 37°C. Injection solutions were prepared as follows: oligomycin (2 μM final concentration), FCCP (1 μM final concentration), and a cocktail containing rotenone (1 μM) and antimycin A (1 μM).

### Cold and thermoneutral challenges

For cold stress, mice were transferred from 21°C to 4°C with free access to water and food. The rectal temperature was measured at the indicated time points. For the thermoneutrality challenge, mice were placed at 30°C with free access to water and food. Body weight, food intake, oxygen consumption, and body temperature were measured.

### Indirect assessment of thermogenesis

This was evaluated by infrared thermography as described [84]. Representative infrared images of mice were measured using a thermal imaging camera (FLIR ONE PRO; FLIR systems, Wilsonville, OR, USA) and analyzed by FLIR Tools software.

### Plasma chemistry

Commercial kits were used to assay plasma triglycerides and insulin (#1488872; Roche Diagnostics, Indianapolis, IN, USA; 80-INSMSU-E01, Alpco Diagnostics, Salem, NH, USA). Plasma glucose was measured with the ALL-IN-ONE blood glucose monitoring system (ACCU-CHEK Compact Plus, Roche Diagnostics). Plasma ALT, AST, CREA, and BUN were measured on a clinical autoanalyzer (Beckman Coulter DX, Brea, CA, USA) in a hospital.

### Proteasomal activity of BAT

This was assayed with a proteasome activity fluorometric assay kit (BioVision, Milpitas, CA, USA) as described [62].

### Primary adipocyte

Primary adipocytes were cultured as described [85–87]. Briefly, iWAT and BAT were taken from 5-h fasted mice. The collected adipose tissue was minced and digested in 0.1% Type I collagenase (Sigma-Aldrich) containing 2% BSA (Sigma-Aldrich) at room temperature for 1 h. The cell suspension was filtered and centrifuged at 2,000 × *g* for 10 min. Cells were then resuspended and cultured in DMEM with 10% FBS (ZETA Life, Menlo Park, CA, USA) at 37°C. Adipocyte differentiation was induced with medium containing 10% FBS, 0.25 mM IBMX, 0.5 μM dexamethasone, 1 μM rosiglitazone, 850 nM insulin, 1 nM T3, and 0.125 mM indomethacin for 48 h. Then, the medium was replaced by one containing 10% FBS, 850 nM insulin, 1 nM T3, and 20 μM UA or DMSO. DMSO was used as the vehicle for different treatments.

### Histology

Portions of liver, BAT, iWAT, and eWAT were fixed in 4% paraformaldehyde. HE staining was performed as described [88]. Immunohistochemistry and immunofluorescence staining of UCP-1 were performed according to the manufacturer’s instructions. In primary cultured adipocytes, immunofluorescence staining of MyHC (R&D Systems, 1:100; Minneapolis, MN, USA) and MyOD (Santa Cruz Biotechnology, 1:100; Dallas, TX, USA) were performed according to the manufacturer’s instructions. The images were acquired using BioTEX Software.

### mtDNA copy number

mtDNA copy number was determined by quantitative Real-time PCR as described [89]. Briefly, total DNA was isolated from the cells using DNeasy Blood & Tissue Kit (Qiagen, 69506; Hilden, Germany) according to the manufacturer’s instructions. The mtDNA copy number was calculated from the ratio of Co1 (mitochondrial-encoded gene)/Ndufv1 (nuclear-encoded gene).

### Deiodinase type 2 activity

The activity of type 2 deiodinase was determined in duplicate in iWAT and BAT homogenates (100 μg protein per 100 μL reaction) as described [90,91].

### T3 and T4 measurements

Levels of T3 and T4 in the circulation, adipose tissue, and cultured adipocytes were quantified by a multiplexed ELISA kit (# EIAT3C; Thermo Fisher Scientific, Waltham, MA, USA) with a Luminex 200 analyzer following the manufacturer’s instructions.

### IL6 and TNFα measurements

Circulating levels of IL-6 and TNF-α were quantified using a multiplexed ELISA kit (Shanghai Enzyme Biotechnology Co., Shanghai, China).

### Real-time qPCR and western blots

qPCR was performed as described [88]. The sequence of primers used is listed in S1 Table. Western blots were performed as described [88]. The information about antibodies used for immunoblotting is listed in S2 Table.

### Skeletal muscle function analysis

Kondziela's inverted screen test was performed as described [92,93]. Briefly, each mouse was put on the center of the wire mesh and rotated to an inverted position. The time when the mouse falls off was measured and scored as the following: 1, falling between 1 and 10 s; 2, between 11 and 25 s; 3, between 26 and 60 s; 4, between 60 and 90 s; 5, after 90 s. The forelimb grip strength test was performed in mice by using a grip strength meter (Columbus Instruments). The antifatigue experiment was performed as described [94]. Briefly, mice were placed in a swimming pool filled with fresh water at 25 ± 1°C. A tin wire (5% of body weight) was loaded on the root of the mouse’s tail. The swimming period was regarded as the time spent by the mouse floating in the water, struggling, until exhausted (failed to rise to the surface of the water within 10 s).

### Statistical analysis

Data are presented as mean ± standard error of the mean. Statistical analysis was evaluated using Student *t* tests. Statistical significance was defined as *p < 0.05, **p < 0.01, and ***p < 0.001 versus controls.

## Supporting information

S1 Fig UA treatment does not prevent weight gain in mice kept under normal diet. (A) Schematic diagram. Six pairs of C57BL/6 male mice fed a normal diet were treated with 30 mg/kg/day of UA or vehicle by gavage starting from 8 weeks of age for a period of 10 weeks. (B) Body weight time course. (C) Daily food intake. (D) Body composition measured by DEXA scan after 5 weeks of treatment. (E) Levels of plasma glucose after 5 h fasting (*n* = 6). The concentration of T3 in (F) BAT and (G) iWAT of normal diet-fed mice with UA or vehicle treatment (*n* = 6). The underlying data for this figure can be found in S1 Data. BAT, brown adipose tissue; DEXA, dual-energy X-ray absorptiometry; iWAT, inguinal white adipose tissue; T3, triiodothyronine; UA, urolithin A.
(TIF)

S2 Fig UA prevents weight gain in *ob*/*ob* mice kept under normal diet. Six-week-old *ob*/*ob* mice kept on a normal diet received UA (30 mg kg^−1^ d^−1^) or vehicle (0.1% Tween 80) for a period of 6 weeks. (A) Weekly body weights of mice. (B) Daily food intake. (C) Weight of total fat and lean mass measured after 6 weeks of UA or vehicle treatment (*n* = 5). (D) The concentration of T3 and T4 in BAT and iWAT of *ob*/*ob* mice with UA or vehicle treatment. (E) mRNA expression of DIO2 in BAT and iWAT (*n* = 5). The underlying data for this figure can be found in S1 Data. BAT, brown adipose tissue; DIO2, Deiodinase 2; iWAT, inguinal white adipose tissue; T3, triiodothyronine; T4, tetraiodothyronine; UA, urolithin A.
(TIF)

S3 Fig The preventive effect of UA and orlistat on HFD-induced obesity. Eight-week-old male C57BL/6 mice were kept under HFD. (A) Weekly body weights of mice. (B) Daily food intake. Internal comparisons were performed between the vehicle and UA groups, as well as the vehicle and orlistat groups. The underlying data for this figure can be found in S1 Data. HFD, high-fat diet; UA, urolithin A.
(TIF)

S4 Fig UA treatment does not cause toxicity in mice. Levels of plasma (A) ALT and (B) AST (*n* = 6). Levels of plasma (C) BUN and (D) CREA (*n* = 6). These parameters were measured after 10 weeks of UA or vehicle treatment in mice under HFD feeding. The underlying data for this figure can be found in S1 Data. ALT, alanine aminotransferase; AST, aspartate aminotransferase; BUN, blood urea nitrogen; CREA, creatinine; HFD, high-fat diet; UA, urolithin A.
(TIF)

S5 Fig The effects of UA on systemic inflammation and liver fat content. Circulating levels of (A) TNF-α and (B) IL-6 in HFD-fed mice with UA or vehicle treatment (*n* = 6). (C) HE staining of liver. (D) Levels of liver TG content. (E) Liver weight of UA- or vehicle-treated mice under HFD feeding (*n* = 6). The underlying data for this figure can be found in S1 Data. HE, hematoxylin–eosin; HFD, high-fat diet; IL-6, interleukin 6; TG, triglyceride; TNF-α, tumor necrosis factor-α; UA, urolithin A.
(TIF)

S6 Fig UA treatment reverses HFD-induced obesity and hyperglycemia. (A) An obese mouse model was induced by 8-week HFD feeding. When the body mass reached around 35 g, UA was given for a period of 10 weeks by gavage. (B) Body weight time course. (C) Body composition measured by DEXA scan after 6 weeks of UA treatment. (D) Daily food intake. (E) Levels of plasma TG after 10 weeks of UA treatment. Levels of (F) plasma glucose and (G) insulin. (H) HOMA-IR. (I) and (J) ITT performed after 8 weeks of UA treatment. (K) and (L) ITT calculated by percentage of glucose changes and the AUC. (M) and (N) GTT performed after 6 weeks of UA treatment. (O) and (P) GTT calculated by changes of glucose and AUC *n* = 6 for each experiment. The underlying data for this figure can be found in S1 Data. AUC, area under the curve; DEXA, dual-energy X-ray absorptiometry; GTT, glucose tolerance test; HFD, high-fat diet; HOMA-IR, homeostasis model assessment-estimated insulin resistance; ITT, insulin tolerance test; TG, triglyceride; UA, urolithin A.
(TIF)

S7 Fig The effect of UA on oxygen consumption on mice receiving UA or vehicle after 7 days. Oxygen consumption was measured by calorimetry in UA-treated and control mice after 7 days of treatment (*n* = 6). The underlying data for this figure can be found in S1 Data. UA, urolithin A.
(TIF)

S8 Fig Adipocyte size distribution of iWAT and eWAT in UA-treated and control mice. Morphometric analysis of adipocyte cell size distribution (based on a total of about 1,000 cells/ treatment) of (A and B) iWAT and of (C and D) eWAT in UA-treated mice and controls (*n* = 3). Adipocyte surfaces are binned at an interval of 250 μm^2^, and data are expressed as percentage of cells per bin. The underlying data for this figure can be found in S1 Data. eWAT, epididymal white adipose tissue; iWAT, inguinal WAT; UA, urolithin A.
(TIF)

S9 Fig Hypothyroidism mouse model induced by PTU treatment. Eight-week-old male C57BL/6 mice were treated with PTU (8.5 mg/100 ml in the drinking water) or vehicle (water) for 2 weeks. (A) Circulating levels of T3. (B) Circulating levels of T4 (*n* = 6). The underlying data for this figure can be found in S1 Data. PTU, propylthiouracil; T3, triiodothyronine; T4, tetraiodothyronine.
(TIF)

S10 Fig The effect of UA on the expression levels of lipid synthesis genes in iWAT. (A) mRNA expressions of PPARγ, Cebpα, and Fas in iWAT of UA-treated and control mice. (B) Protein levels of Cebpα, Pparγ, and Fas in iWAT of UA-treated and control mice shown by western blot. The underlying data for this figure can be found in S1 Data. Cebpα, CCAAT enhancer binding protein α; Fas, Fatty acid synthase; iWAT, inguinal white adipose tissue; PPARγ, Peroxisome proliferator-activated receptor γ; UA, urolithin A.
(TIF)

S11 Fig UA treatment inhibits primary beige and brown adipocyte differentiation. (A) Schematic diagram of cell treatment. Primary beige and brown adipocytes were differentiated into adipocytes and treated with UA (20 μM) from day 1 to day 7. (B) Oil-Red-O staining of primary beige adipocyte differentiated for 7 days with or without UA treatment. (C) The mRNA and (D) protein levels of UCP-1, PPARγ, FAS, AP2, and adiponectin in beige adipocytes at indicated time points. (E) The relative concentration of T3 in beige adipocytes with or without UA treatment. (F) Oil-Red-O staining of primary brown adipocytes differentiated for 7 days with or without UA treatment. (G) The mRNA and (H) protein expression levels of UCP-1, PPARγ, FAS, AP2, and adiponectin in brown adipocytes at indicated timepoints. (I) The relative concentration of T3 in brown adipocytes with or without UA treatment. The underlying data for this figure can be found in S1 Data. AP2, adipocyte protein 2; FAS, Fatty acid synthase; PPARγ, Peroxisome proliferator-activated receptor γ; T3, triiodothyronine; UA, urolithin A; UCP-1, Uncoupling protein 1.
(TIF)

S12 Fig UA treatment does not affect UCP1-independent and β-AR–independent thermogenic pathways. (A) Proteasome activity of BAT in mice under room temperature (21°C) conditions. (B) mRNA expression of the Ca2+ cycling thermogenic markers in iWAT of mice under room temperature (21°C) and thermoneutral (30°C) conditions. (C) mRNA expressions of the creatine cycling thermogenic genes in iWAT of mice under room temperature (23°C) and thermoneutral (30°C) conditions. (D) Immunofluorescent staining of MyoD in the iWAT-derived SVF cells treated with UA or DMSO. (E) Immunofluorescent staining of MyHC in differentiated SVFs treated with UA or DMSO. (F) mRNA expression of the glycolytic beige fat formation marker in differentiated SVFs treated with UA or DMSO. The underlying data for this figure can be found in S1 Data. BAT, brown adipose tissue; iWAT, inguinal white adipose tissue; MyHC, myosin heavy chain; MyoD, myoblast determination protein; SVF, stromal vascular fraction; UA, urolithin A; UCP-1, Uncoupling protein 1; β-AR, β-adrenergic receptor.
(TIF)

S13 Fig UA treatment improves exercise capacity in mice. (A) The gross appearance of skeletal muscle after UA treatment. (B) Kondziela's inverted screen test after UA treatment. (C) Grip strength test. Forelimb (2 paws) grip force measurements after UA treatment. (D) Effect of the UA on the weight-bearing swimming time in mice. The underlying data for this figure can be found in S1 Data. UA, urolithin A.
(TIF)

S1 Raw Image Original blot. (TIF)

S2 Raw Image Original blot. (TIF)

S1 Table Primers used for gene amplification. (DOCX)

S2 Table Antibody used for western blot. (DOCX)

S1 Data Contains underlying data for Figs 1B–1P; 2A–2G, 2I–2K; 3A, 3D, 3E, 3I, 3J; 4B–4F, 4H–4K; 5A–5P; 6A, 6B–6K; 7B–7J; S1B–S1G; S2A–S2E; S3A and S3B; S4A–S4D; S5A, S5B, S5D and S5E; S6B–S6P; S7 and S8A–S8D; S9A and S9B; S10A and S11D, S11E, S11H and S11I; S12A–S12C; S12E and S12F; S13B–S13D Figs. (XLSX)

## Abbreviations

Accβ: Acetyl-CoA carboxylase beta
ALT: alanine aminotransferase
AST: aspartate aminotransferase
BAT: brown adipose tissue
BUN: blood urea nitrogen
Cidea: Cell death inducing DFFA like effector a
Cox7a1: Cytochrome c oxidase subunit 7a1
Cpt1b: Carnitine palmitoyltransferase 1B
CREA: creatinine
Cyt c: Cytochrome c
Cebpα: CCAAT enhancer binding protein α
Dio2: Deiodinase 2
EA: ellagic acid
EE: energy expenditure
Errα: Estrogen related receptor α
ET: ellagitannin
eWAT: epididymal WAT
FDA: Food and Drug Administration
GDF-15: Growth differentiation factor 15
GEO: Gene Expression Omnibus
GIR: glucose infusion rate
GRAS: generally recognized as safe
GTT: glucose tolerance test
HFD: high-fat diet
HOMA-IR: homeostasis model assessment-estimated insulin resistance
IL-6: interleukin 6
iso-UA: iso-urolithin A
ITT: insulin tolerance test
iWAT: inguinal WAT
KEGG: Kyoto Encyclopedia of Genes and Genomes
Ldhβ: Lactate dehydrogenase B
LPS: lipopolysaccharide
mtDNA: mitochondrial DNA
NE: norepinephrine
OCR: oxygen consumption rate
Pcg1α: Peroxisome proliferator-activated receptor gamma coactivator 1α
PPARγ: Peroxisome proliferator-activated receptor γ
PTU: propylthiouracil
RER: respiratory exchange ratio
SNS: sympathetic nervous system
TG: triglyceride
TH: thyroid hormone
TNF-α: tumor necrosis factor-α
T3: triiodothyronine
T4: tetraiodothyronine
UA: urolithin A
UB: urolithin B
UC: urolithin C
UCP-1: Uncoupling protein 1
UD: urolithin D
WAT: white adipose tissue
6-OHDA: 6-hydroxydopamine
β-AR: β-adrenergic receptor
