# Supplementary figures and images for: Urolithin A exerts antiobesity effects through enhancing adipose tissue thermogenesis in mice
Source: PLoS Biol. 2020 Mar 27;18(3):e3000688. doi: 10.1371/journal.pbio.3000688 (PMC7141696; doi:10.1371/journal.pbio.3000688)

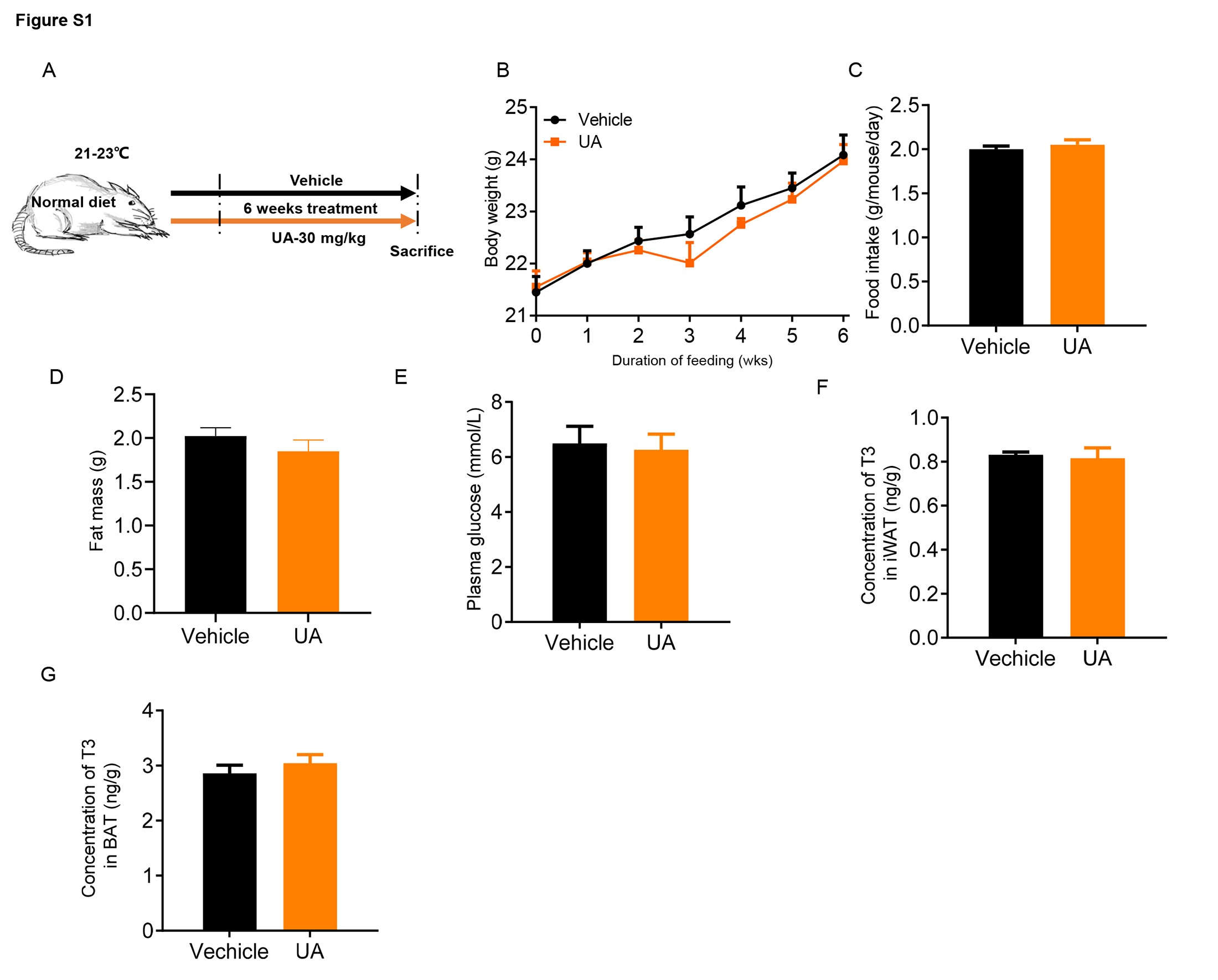

Supplement: S1 Fig — (A) Schematic diagram. Six pairs of C57BL/6 male mice fed a normal diet were treated with 30 mg/kg/day of UA or vehicle by gavage starting from 8 weeks of age for a period of 10 weeks. (B) Body weight time course. (C) Daily food intake. (D) Body composition measured by DEXA scan after 5 weeks of treatment. (E) Levels of plasma glucose after 5 h fasting (n = 6). The concentration of T3 in (F) BAT and (G) iWAT of normal diet-fed mice with UA or vehicle treatment (n = 6). The underlying data for this figure can be found in S1 Data. BAT, brown adipose tissue; DEXA, dual-energy X-ray absorptiometry; iWAT, inguinal white adipose tissue; T3, triiodothyronine; UA, urolithin A. (TIF) [file pbio.3000688.s001.tif]

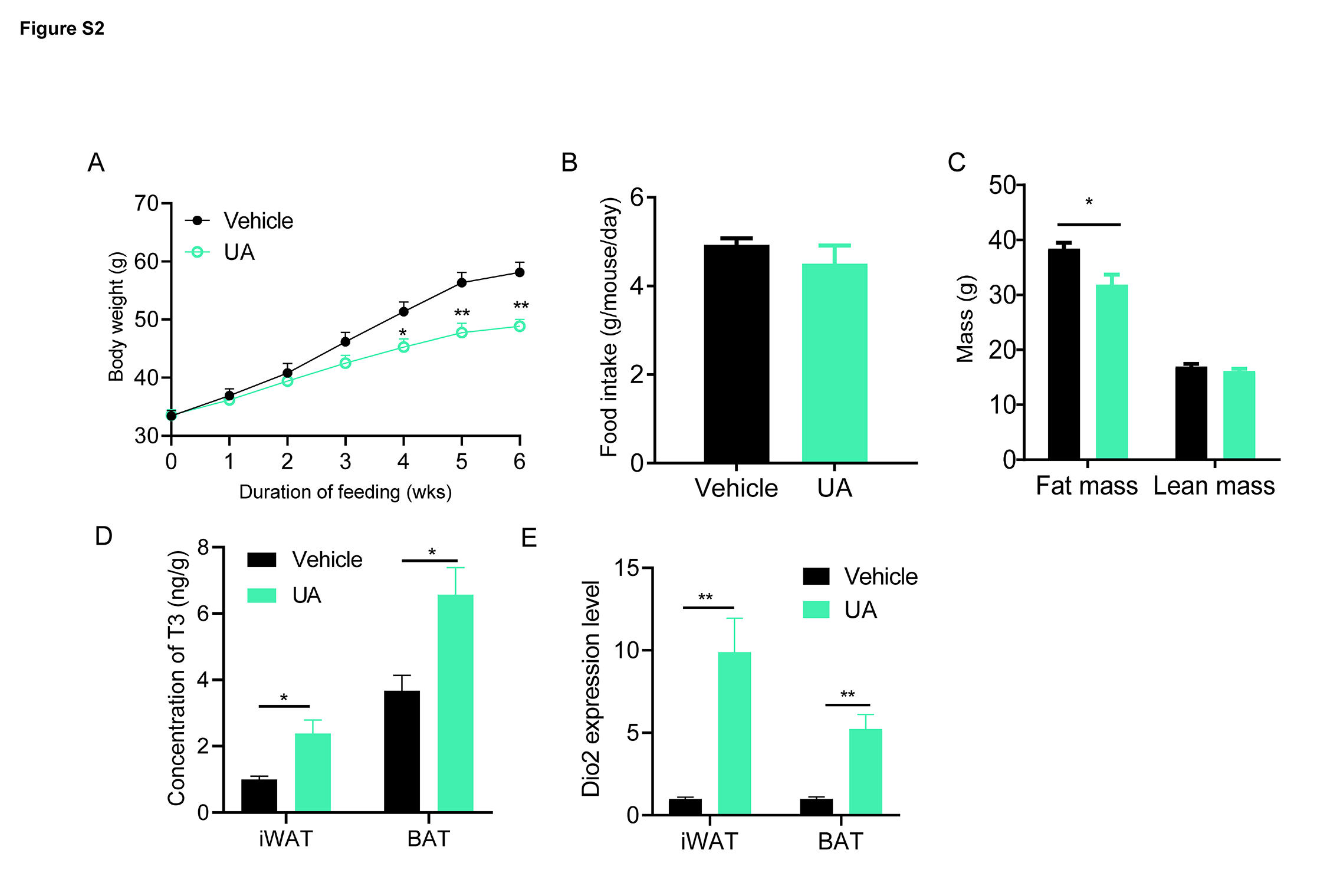

Supplement: S2 Fig — Six-week-old ob/ob mice kept on a normal diet received UA (30 mg kg−1 d−1) or vehicle (0.1% Tween 80) for a period of 6 weeks. (A) Weekly body weights of mice. (B) Daily food intake. (C) Weight of total fat and lean mass measured after 6 weeks of UA or vehicle treatment (n = 5). (D) The concentration of T3 and T4 in BAT and iWAT of ob/ob mice with UA or vehicle treatment. (E) mRNA expression of DIO2 in BAT and iWAT (n = 5). The underlying data for this figure can be found in S1 Data. BAT, brown adipose tissue; DIO2, Deiodinase 2; iWAT, inguinal white adipose tissue; T3, triiodothyronine; T4, tetraiodothyronine; UA, urolithin A. (TIF) [file pbio.3000688.s002.tif]

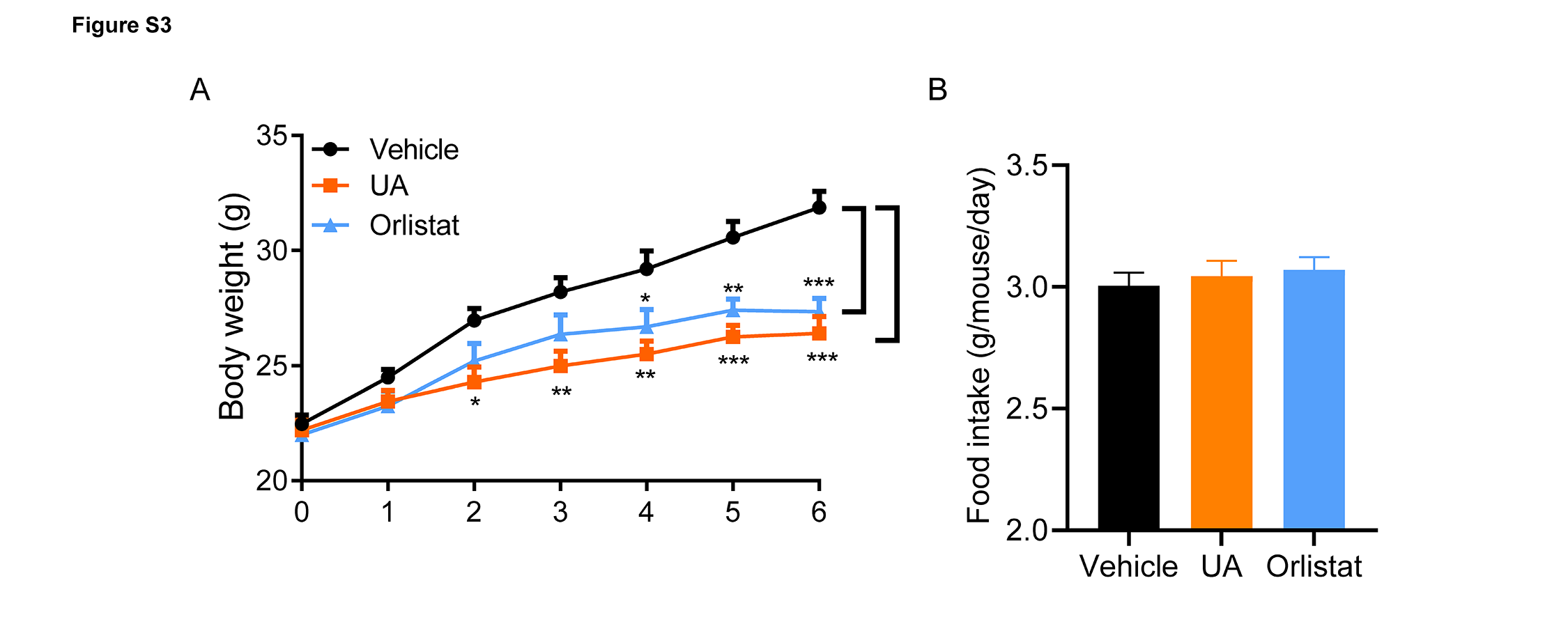

Supplement: S3 Fig — Eight-week-old male C57BL/6 mice were kept under HFD. (A) Weekly body weights of mice. (B) Daily food intake. Internal comparisons were performed between the vehicle and UA groups, as well as the vehicle and orlistat groups. The underlying data for this figure can be found in S1 Data. HFD, high-fat diet; UA, urolithin A. (TIF) [file pbio.3000688.s003.tif]

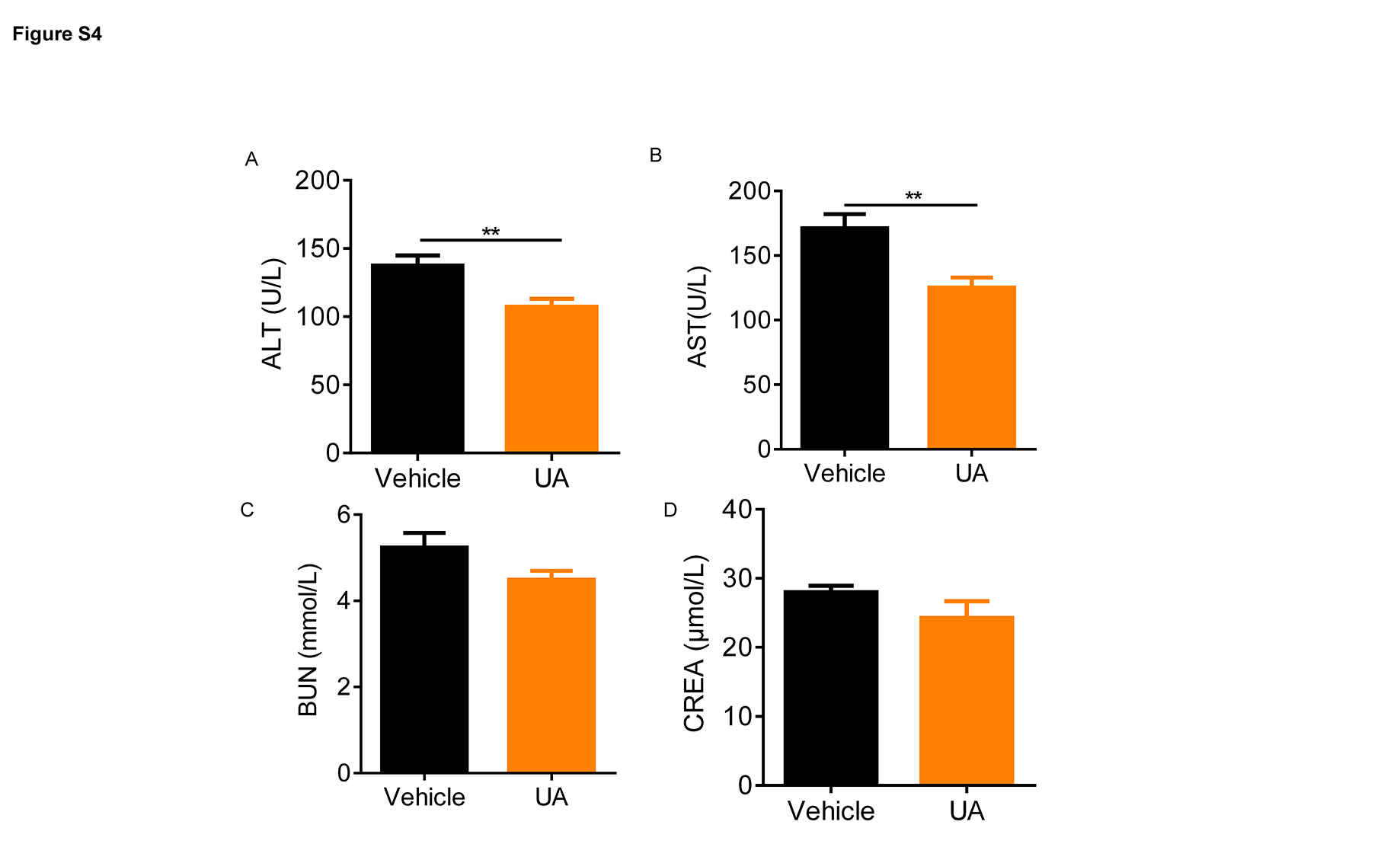

Supplement: S4 Fig — Levels of plasma (A) ALT and (B) AST (n = 6). Levels of plasma (C) BUN and (D) CREA (n = 6). These parameters were measured after 10 weeks of UA or vehicle treatment in mice under HFD feeding. The underlying data for this figure can be found in S1 Data. ALT, alanine aminotransferase; AST, aspartate aminotransferase; BUN, blood urea nitrogen; CREA, creatinine; HFD, high-fat diet; UA, urolithin A. (TIF) [file pbio.3000688.s004.tif]

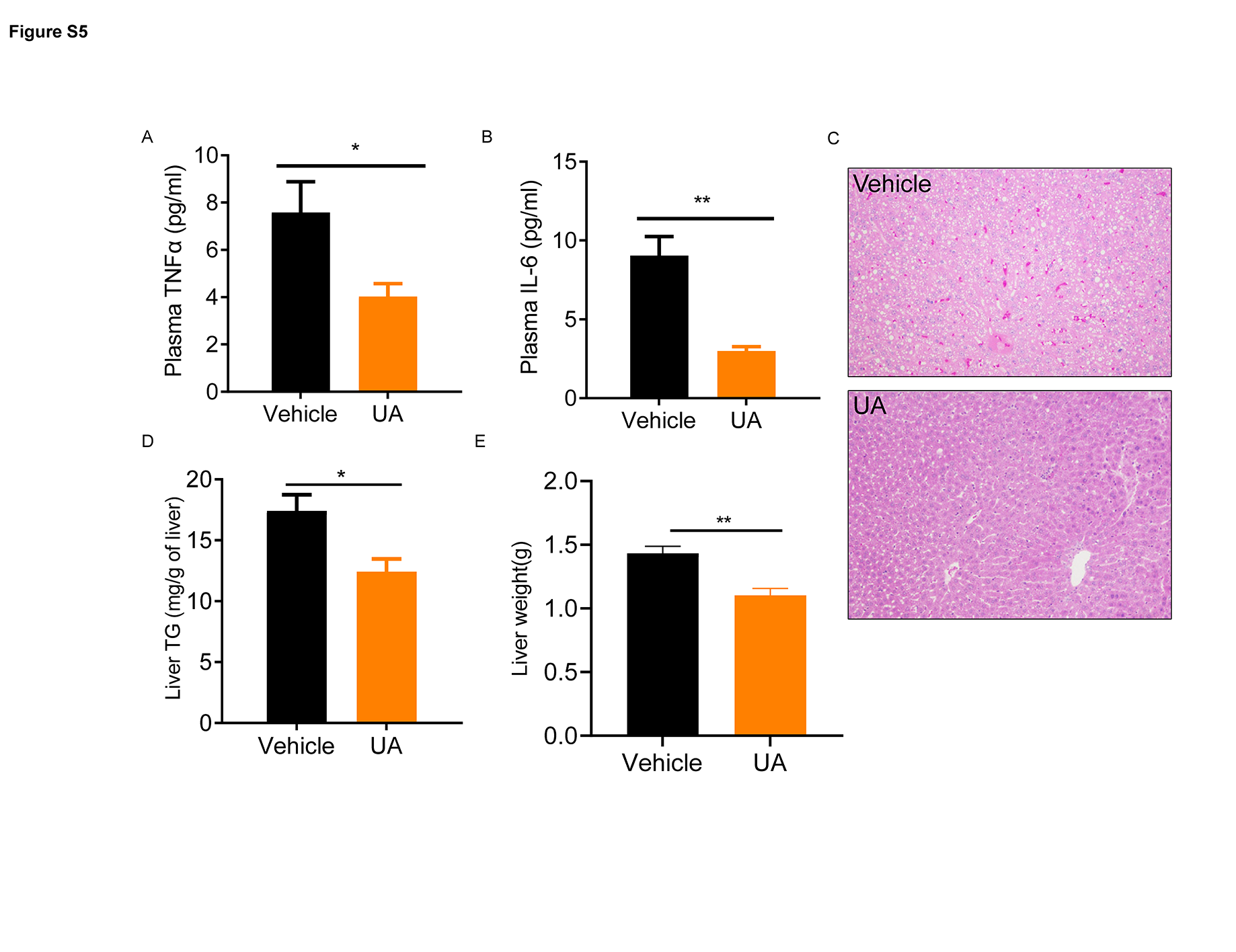

Supplement: S5 Fig — Circulating levels of (A) TNF-α and (B) IL-6 in HFD-fed mice with UA or vehicle treatment (n = 6). (C) HE staining of liver. (D) Levels of liver TG content. (E) Liver weight of UA- or vehicle-treated mice under HFD feeding (n = 6). The underlying data for this figure can be found in S1 Data. HE, hematoxylin–eosin; HFD, high-fat diet; IL-6, interleukin 6; TG, triglyceride; TNF-α, tumor necrosis factor-α; UA, urolithin A. (TIF) [file pbio.3000688.s005.tif]

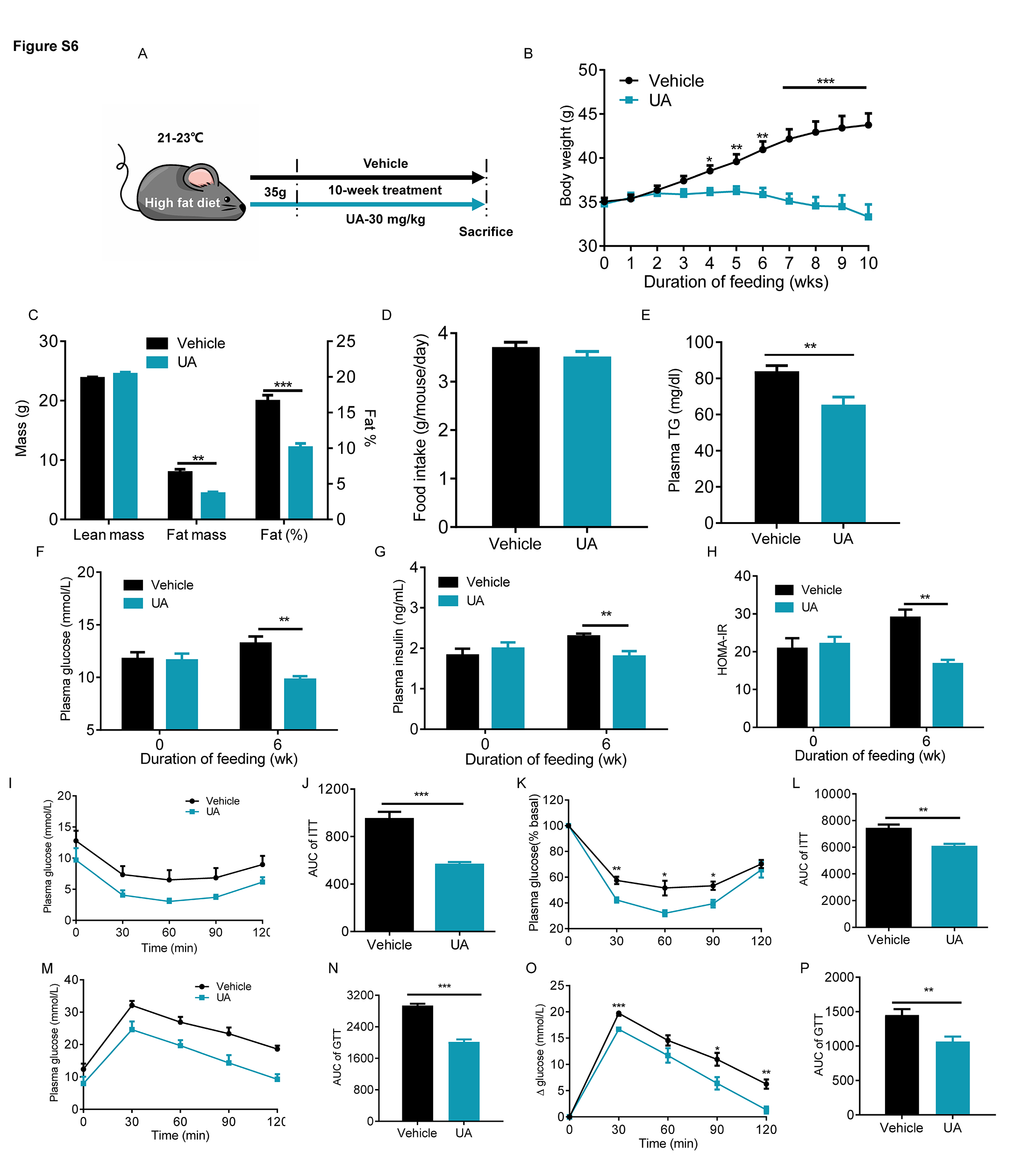

Supplement: S6 Fig — (A) An obese mouse model was induced by 8-week HFD feeding. When the body mass reached around 35 g, UA was given for a period of 10 weeks by gavage. (B) Body weight time course. (C) Body composition measured by DEXA scan after 6 weeks of UA treatment. (D) Daily food intake. (E) Levels of plasma TG after 10 weeks of UA treatment. Levels of (F) plasma glucose and (G) insulin. (H) HOMA-IR. (I) and (J) ITT performed after 8 weeks of UA treatment. (K) and (L) ITT calculated by percentage of glucose changes and the AUC. (M) and (N) GTT performed after 6 weeks of UA treatment. (O) and (P) GTT calculated by changes of glucose and AUC n = 6 for each experiment. The underlying data for this figure can be found in S1 Data. AUC, area under the curve; DEXA, dual-energy X-ray absorptiometry; GTT, glucose tolerance test; HFD, high-fat diet; HOMA-IR, homeostasis model assessment-estimated insulin resistance; ITT, insulin tolerance test; TG, triglyceride; UA, urolithin A. (TIF) [file pbio.3000688.s006.tif]

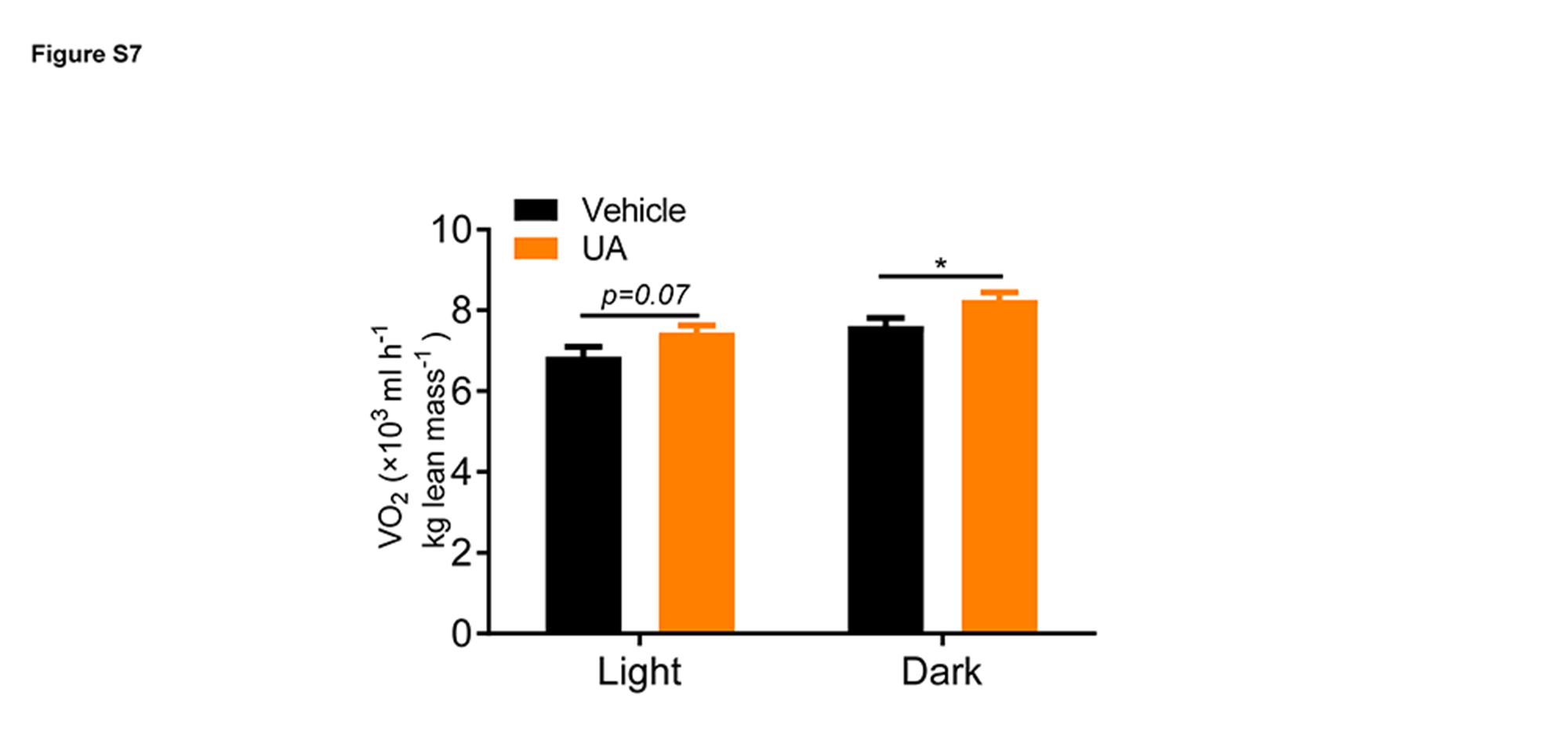

Supplement: S7 Fig — Oxygen consumption was measured by calorimetry in UA-treated and control mice after 7 days of treatment (n = 6). The underlying data for this figure can be found in S1 Data. UA, urolithin A. (TIF) [file pbio.3000688.s007.tif]

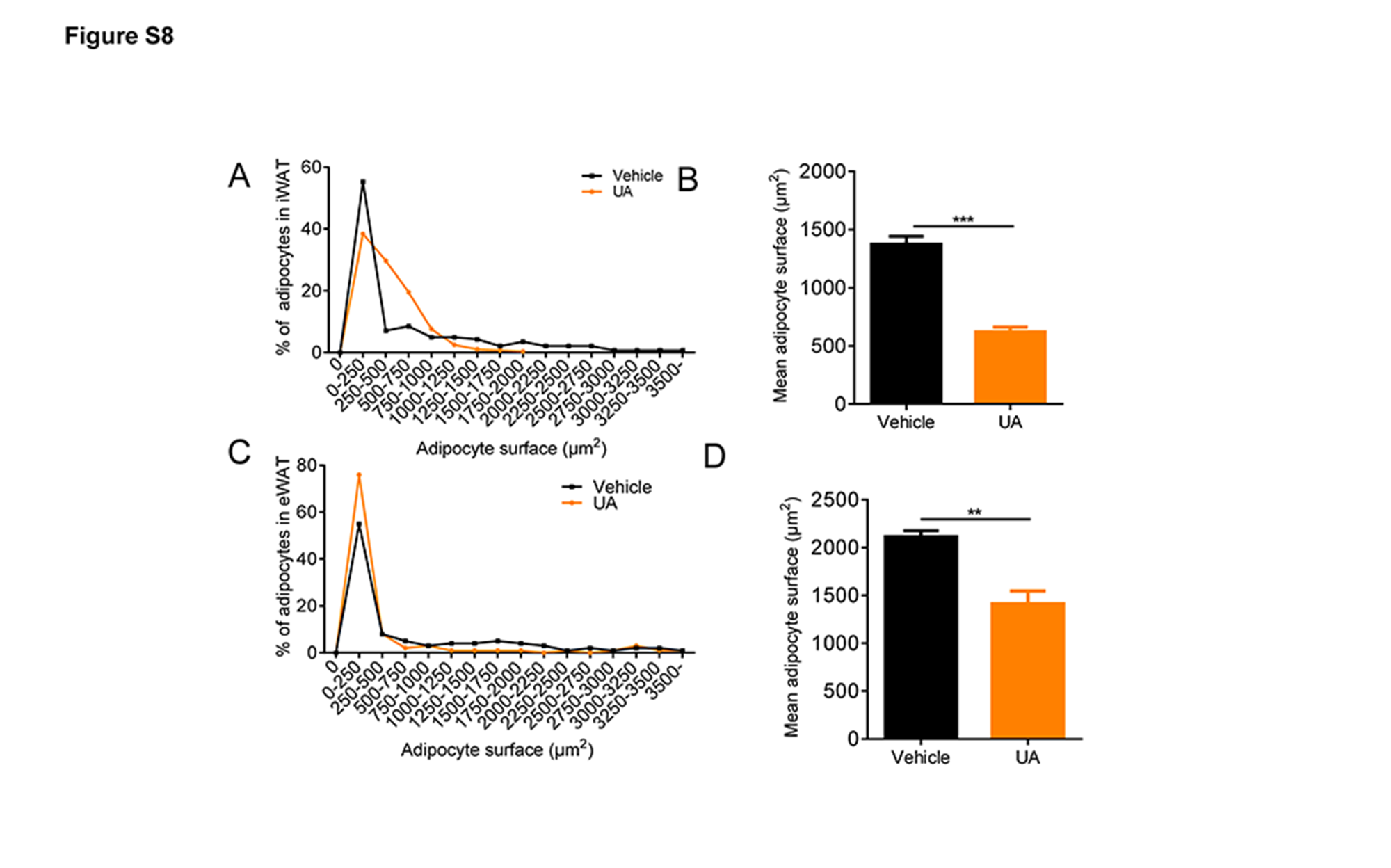

Supplement: S8 Fig — Morphometric analysis of adipocyte cell size distribution (based on a total of about 1,000 cells/ treatment) of (A and B) iWAT and of (C and D) eWAT in UA-treated mice and controls (n = 3). Adipocyte surfaces are binned at an interval of 250 μm2, and data are expressed as percentage of cells per bin. The underlying data for this figure can be found in S1 Data. eWAT, epididymal white adipose tissue; iWAT, inguinal WAT; UA, urolithin A. (TIF) [file pbio.3000688.s008.tif]

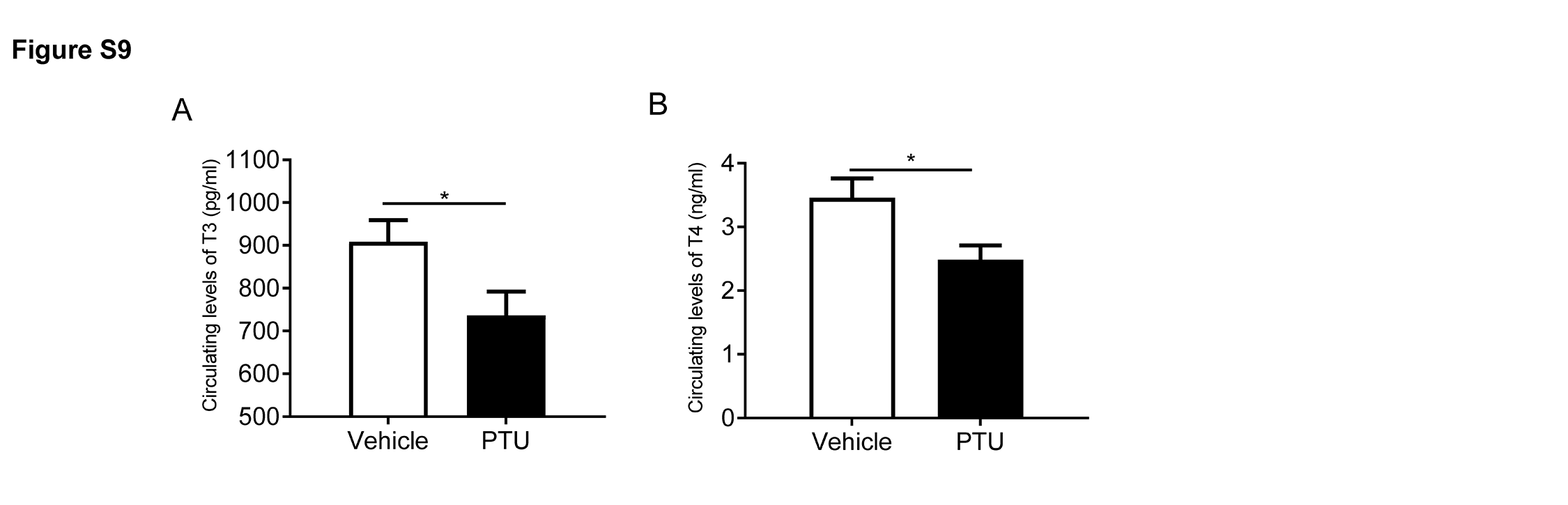

Supplement: S9 Fig — Eight-week-old male C57BL/6 mice were treated with PTU (8.5 mg/100 ml in the drinking water) or vehicle (water) for 2 weeks. (A) Circulating levels of T3. (B) Circulating levels of T4 (n = 6). The underlying data for this figure can be found in S1 Data. PTU, propylthiouracil; T3, triiodothyronine; T4, tetraiodothyronine. (TIF) [file pbio.3000688.s009.tif]

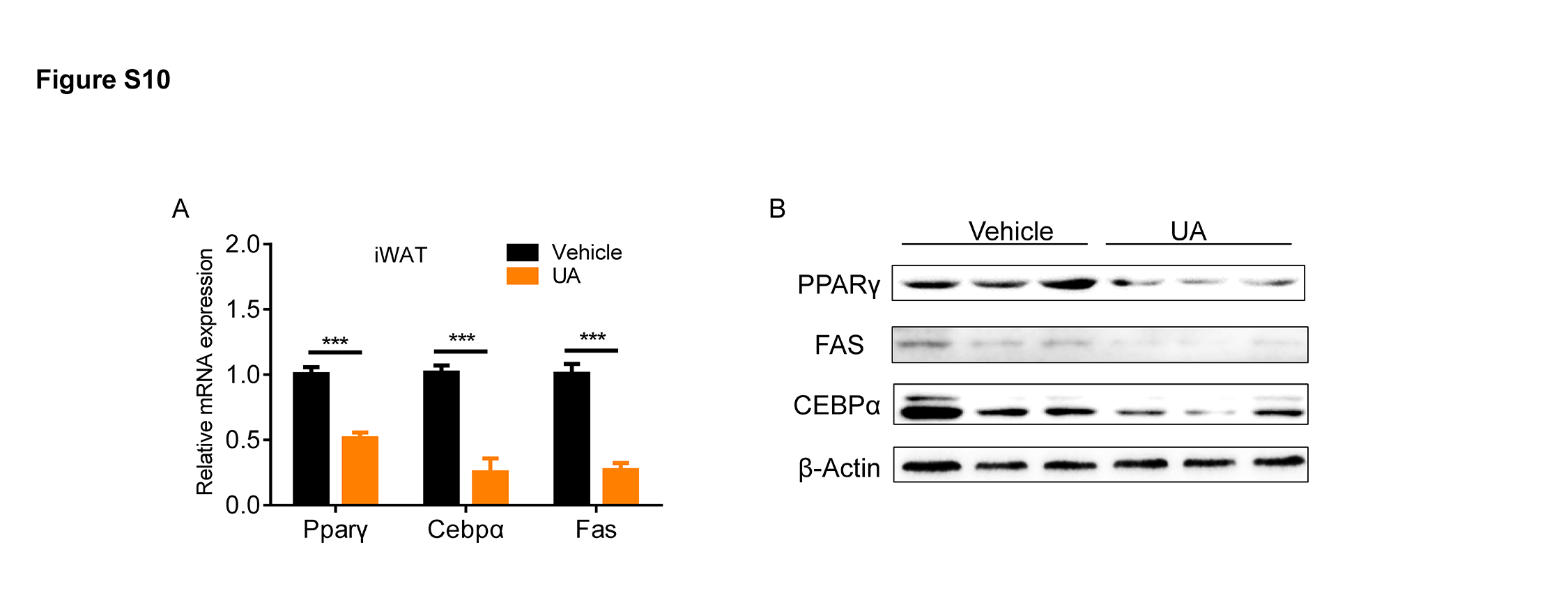

Supplement: S10 Fig — (A) mRNA expressions of PPARγ, Cebpα, and Fas in iWAT of UA-treated and control mice. (B) Protein levels of Cebpα, Pparγ, and Fas in iWAT of UA-treated and control mice shown by western blot. The underlying data for this figure can be found in S1 Data. Cebpα, CCAAT enhancer binding protein α; Fas, Fatty acid synthase; iWAT, inguinal white adipose tissue; PPARγ, Peroxisome proliferator-activated receptor γ; UA, urolithin A. (TIF) [file pbio.3000688.s010.tif]

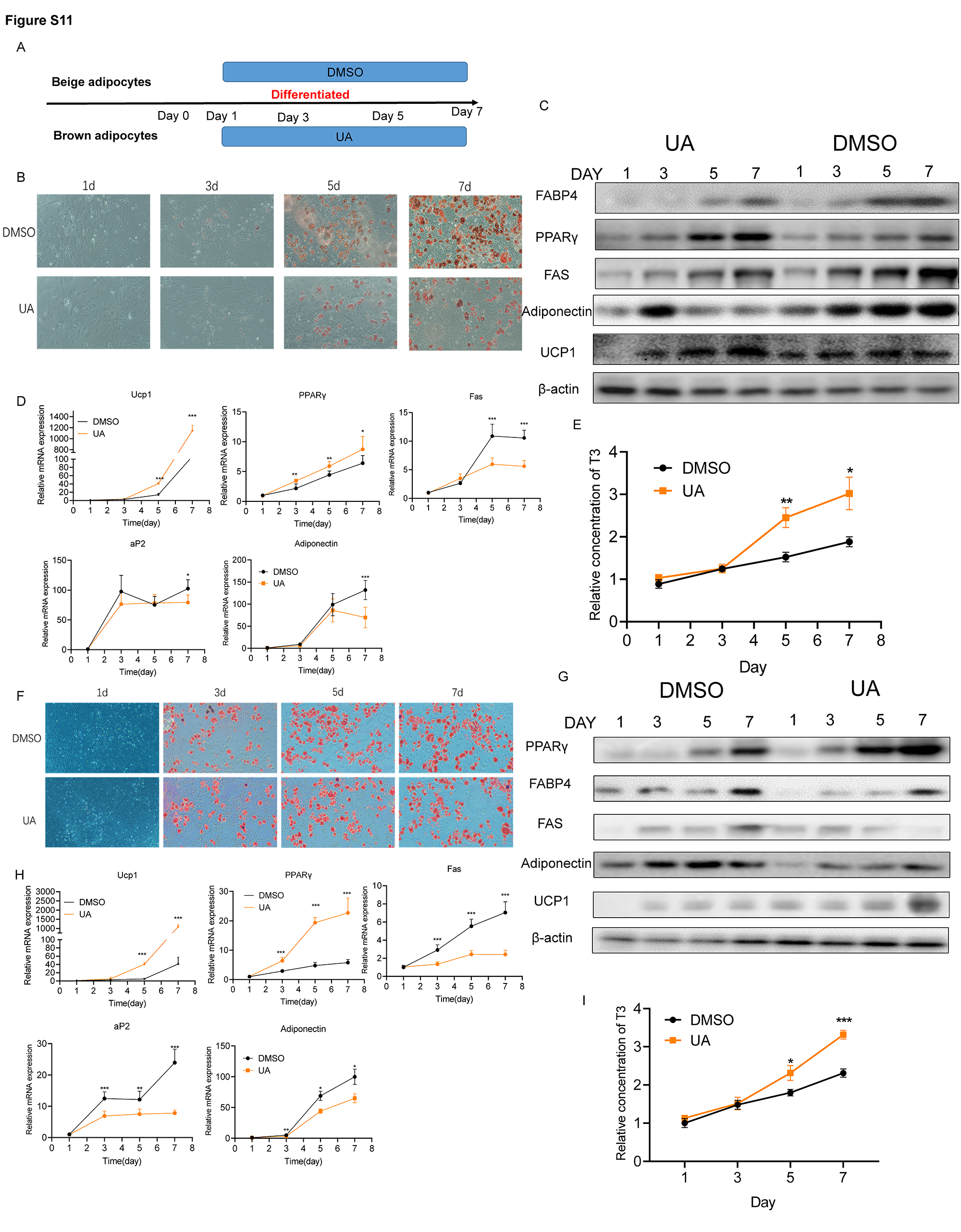

Supplement: S11 Fig — (A) Schematic diagram of cell treatment. Primary beige and brown adipocytes were differentiated into adipocytes and treated with UA (20 μM) from day 1 to day 7. (B) Oil-Red-O staining of primary beige adipocyte differentiated for 7 days with or without UA treatment. (C) The mRNA and (D) protein levels of UCP-1, PPARγ, FAS, AP2, and adiponectin in beige adipocytes at indicated time points. (E) The relative concentration of T3 in beige adipocytes with or without UA treatment. (F) Oil-Red-O staining of primary brown adipocytes differentiated for 7 days with or without UA treatment. (G) The mRNA and (H) protein expression levels of UCP-1, PPARγ, FAS, AP2, and adiponectin in brown adipocytes at indicated timepoints. (I) The relative concentration of T3 in brown adipocytes with or without UA treatment. The underlying data for this figure can be found in S1 Data. AP2, adipocyte protein 2; FAS, Fatty acid synthase; PPARγ, Peroxisome proliferator-activated receptor γ; T3, triiodothyronine; UA, urolithin A; UCP-1, Uncoupling protein 1. (TIF) [file pbio.3000688.s011.tif]

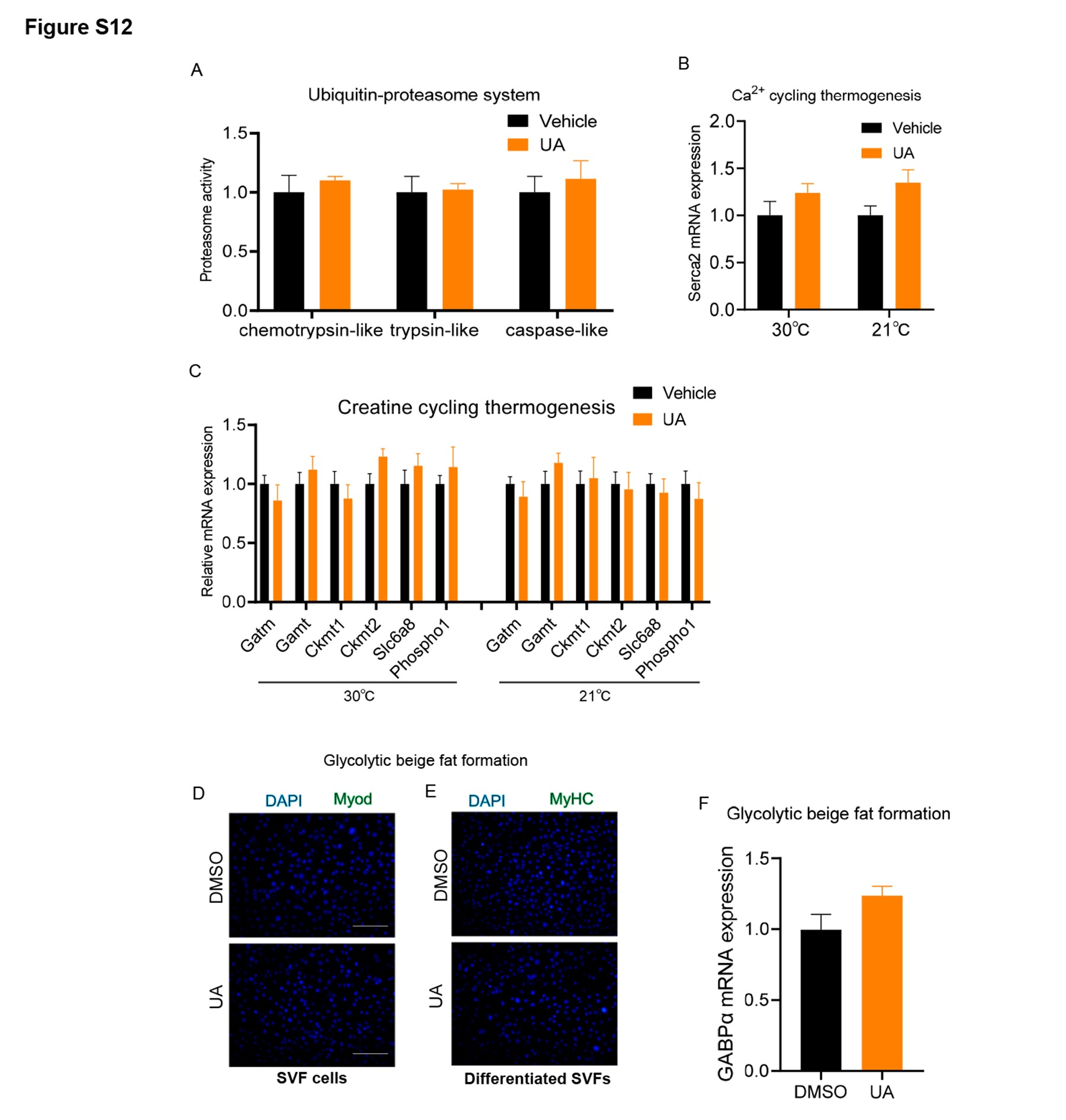

Supplement: S12 Fig — (A) Proteasome activity of BAT in mice under room temperature (21°C) conditions. (B) mRNA expression of the Ca2+ cycling thermogenic markers in iWAT of mice under room temperature (21°C) and thermoneutral (30°C) conditions. (C) mRNA expressions of the creatine cycling thermogenic genes in iWAT of mice under room temperature (23°C) and thermoneutral (30°C) conditions. (D) Immunofluorescent staining of MyoD in the iWAT-derived SVF cells treated with UA or DMSO. (E) Immunofluorescent staining of MyHC in differentiated SVFs treated with UA or DMSO. (F) mRNA expression of the glycolytic beige fat formation marker in differentiated SVFs treated with UA or DMSO. The underlying data for this figure can be found in S1 Data. BAT, brown adipose tissue; iWAT, inguinal white adipose tissue; MyHC, myosin heavy chain; MyoD, myoblast determination protein; SVF, stromal vascular fraction; UA, urolithin A; UCP-1, Uncoupling protein 1; β-AR, β-adrenergic receptor. (TIF) [file pbio.3000688.s012.tif]

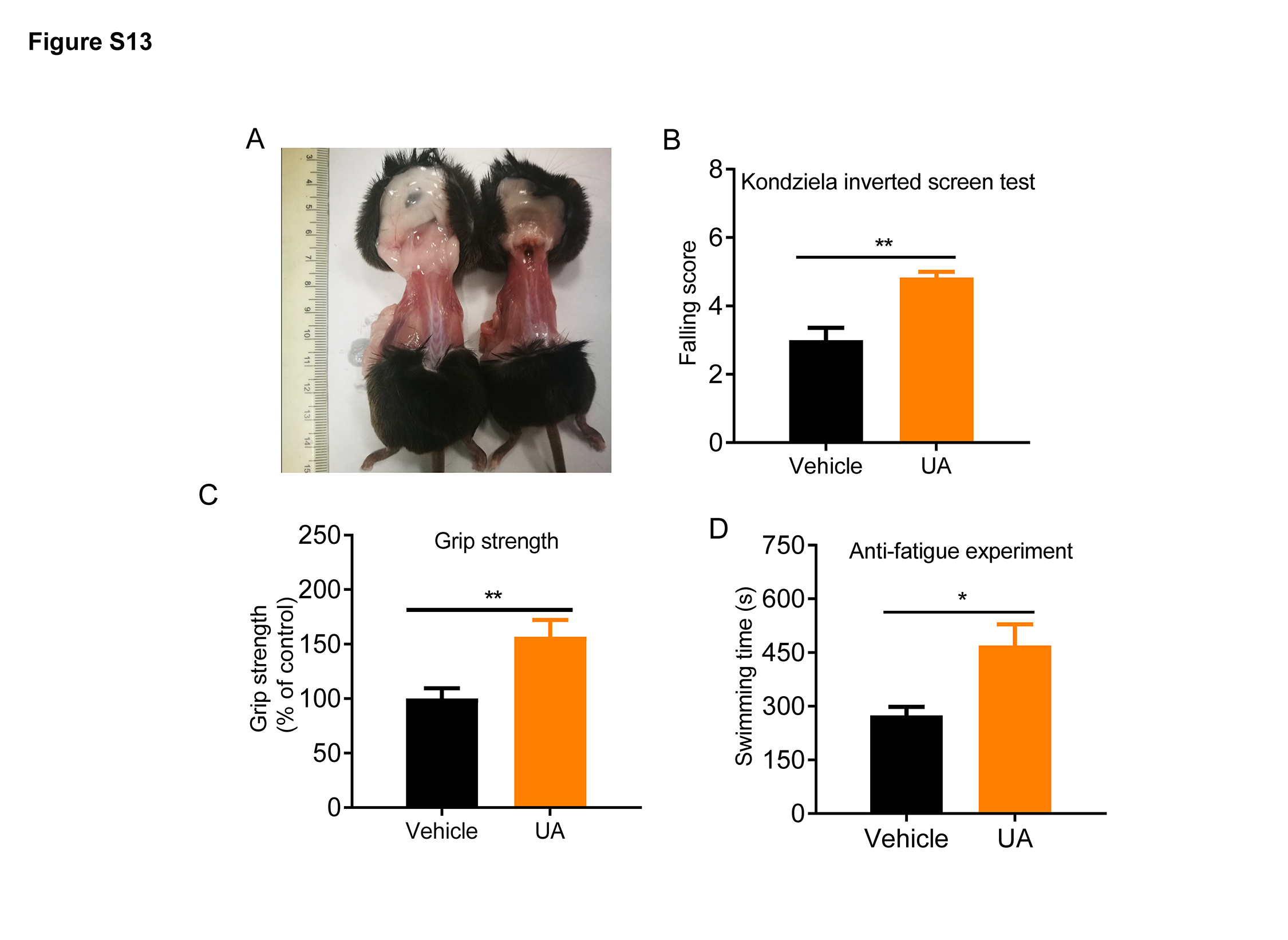

Supplement: S13 Fig — (A) The gross appearance of skeletal muscle after UA treatment. (B) Kondziela's inverted screen test after UA treatment. (C) Grip strength test. Forelimb (2 paws) grip force measurements after UA treatment. (D) Effect of the UA on the weight-bearing swimming time in mice. The underlying data for this figure can be found in S1 Data. UA, urolithin A. (TIF) [file pbio.3000688.s013.tif]

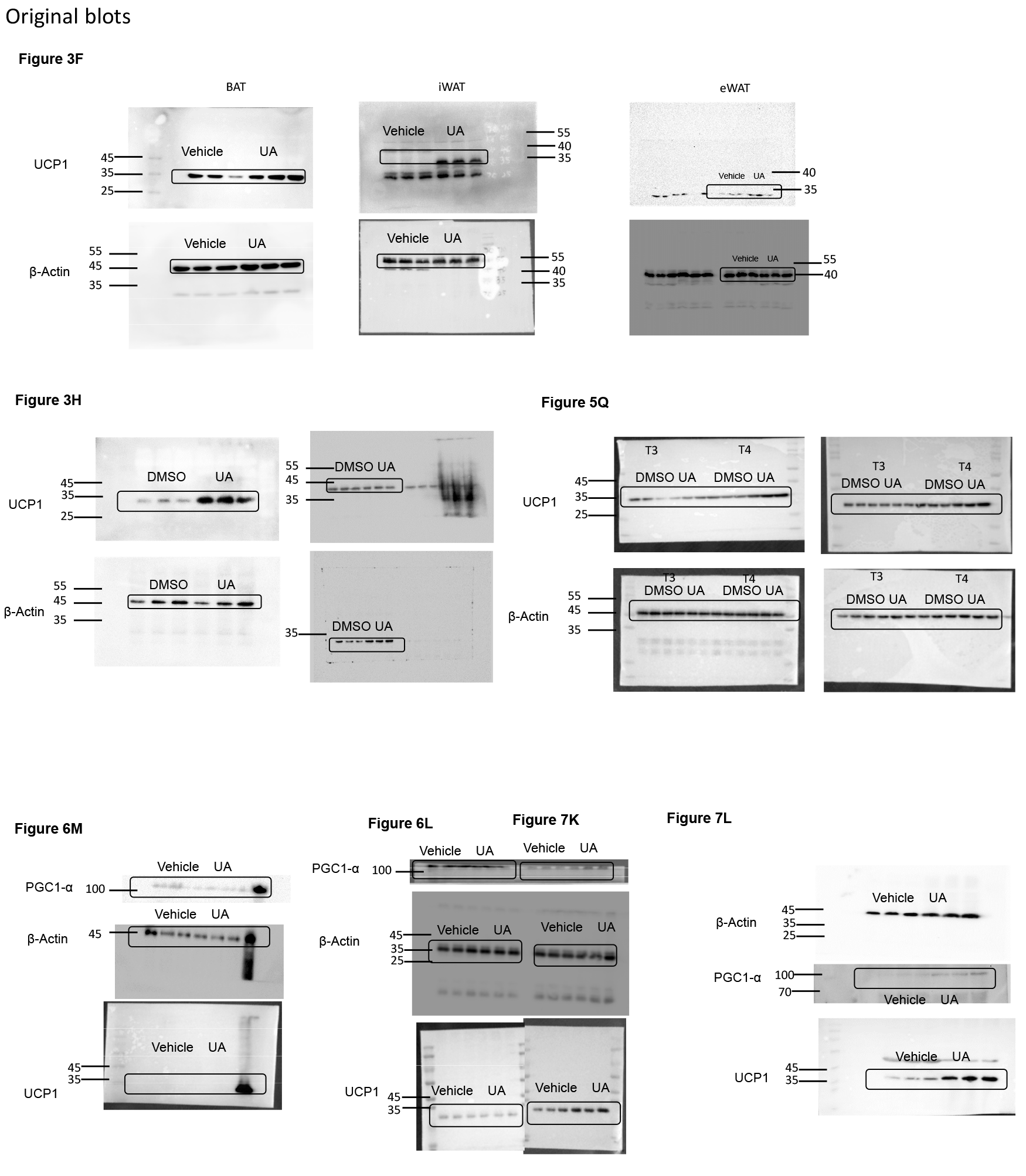

Supplement: S1 Raw Image — (TIF) [file pbio.3000688.s014.tif]

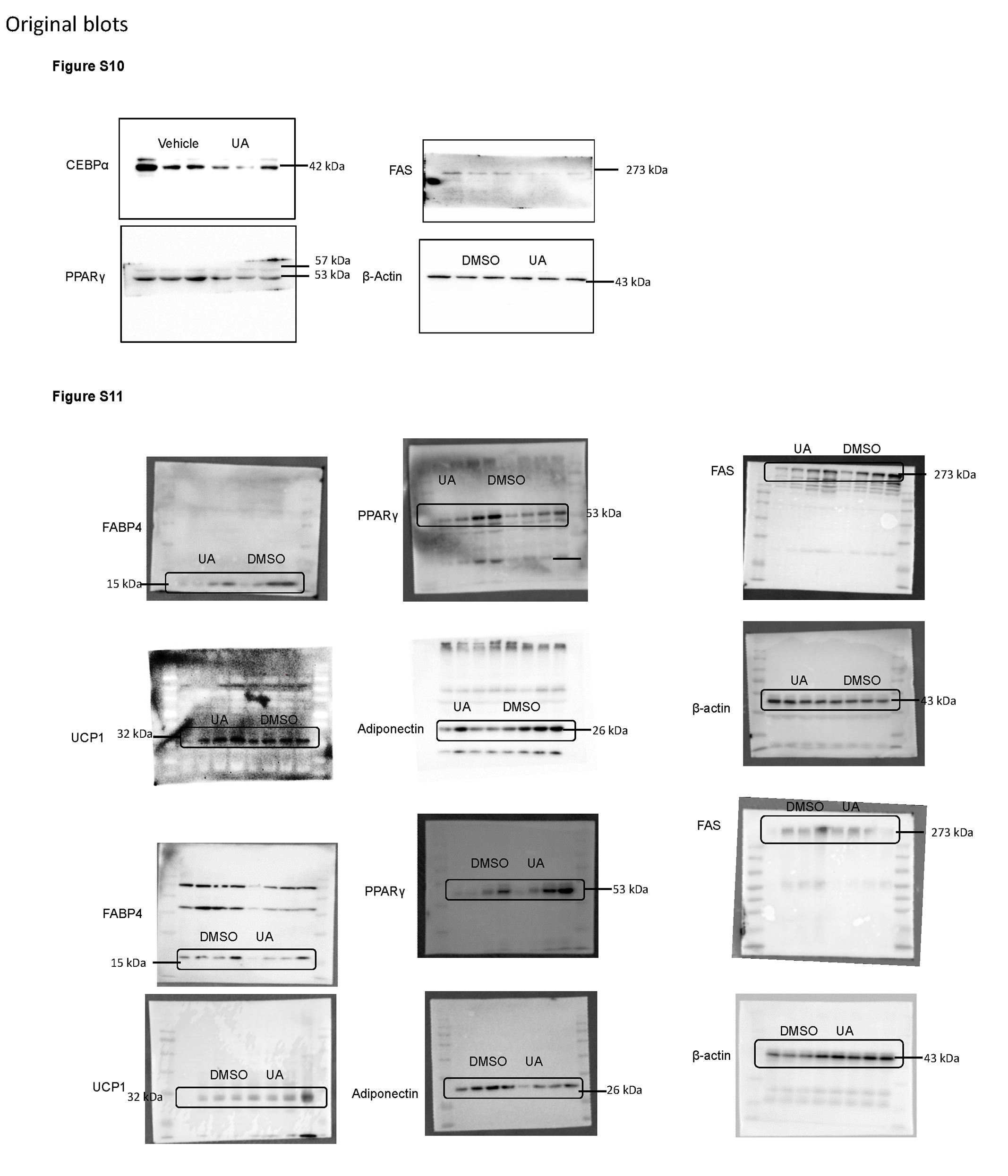

Supplement: S2 Raw Image — (TIF) [file pbio.3000688.s015.tif]

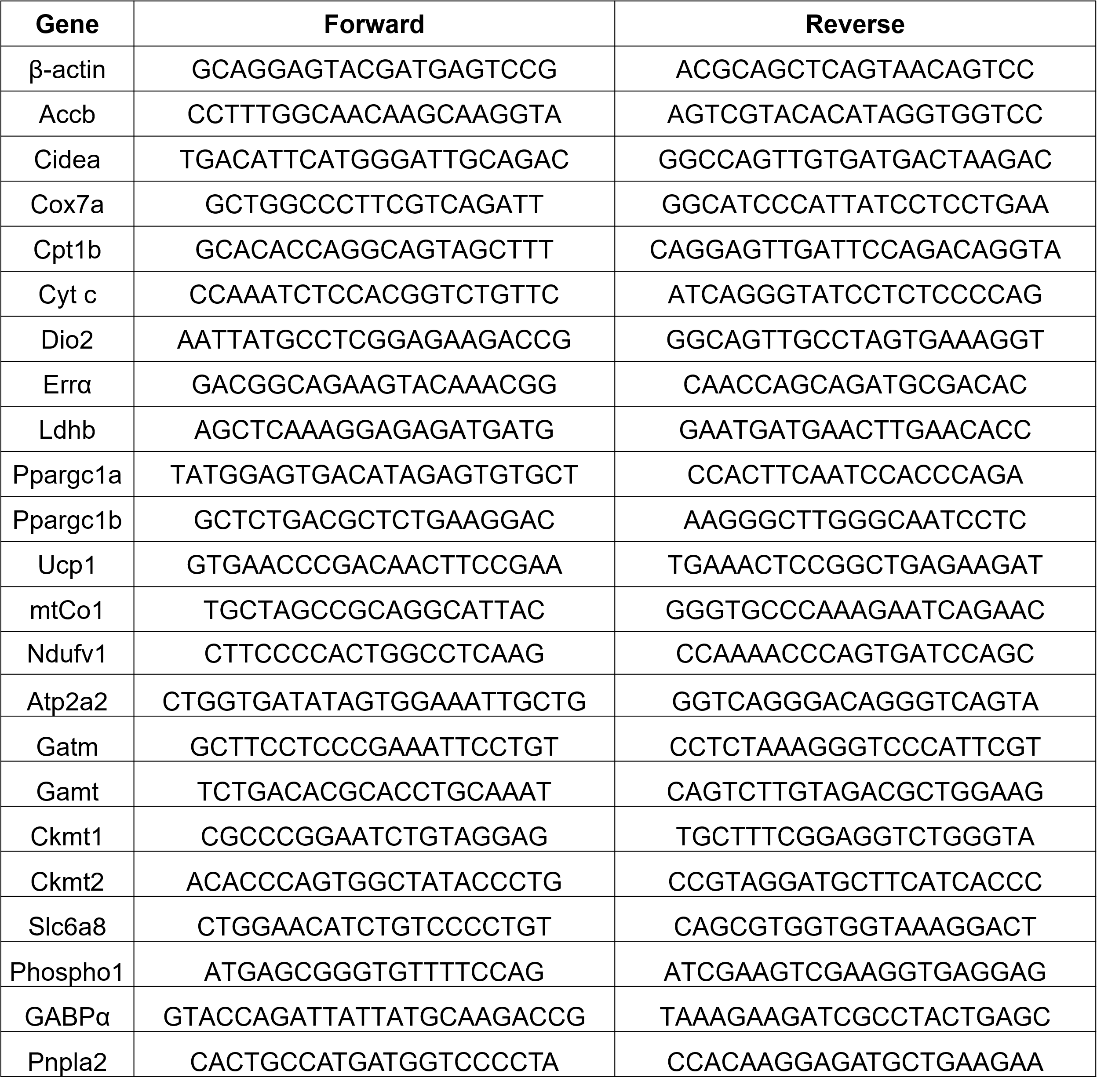


**S1 Table. Primers used for gene amplification.**

Supplement: S1 Table — (DOCX) [file pbio.3000688.s016.docx]

**Table S2. Antibody used for Western blot.**
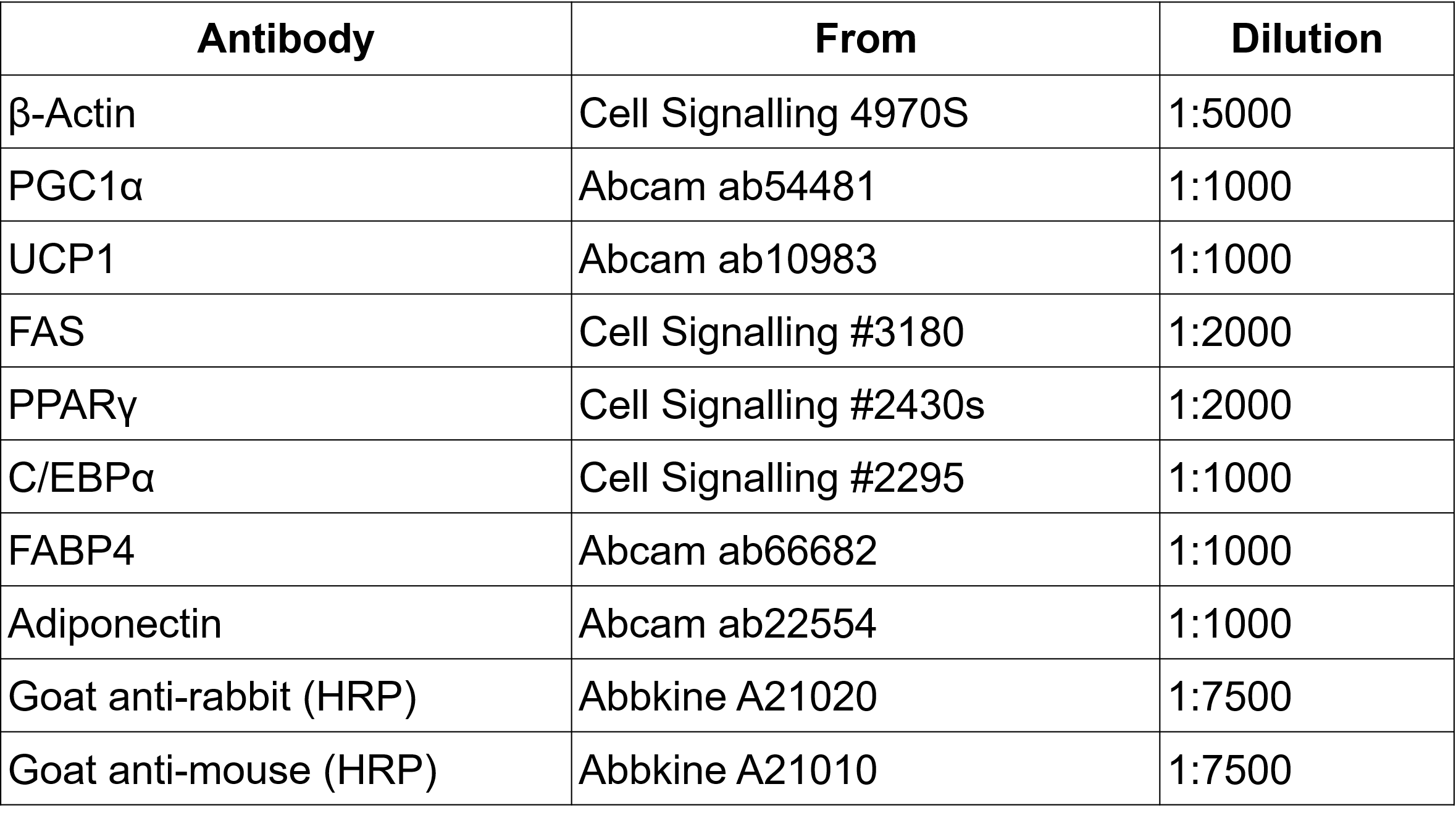

Supplement: S2 Table — (DOCX) [file pbio.3000688.s017.docx]
